# The Influence of Trinucleotide Repeats in the Androgen Receptor Gene and Testosterone Level on Circulating Proteins in Male Participants: Proteomics Analysis Using the UK Biobank Data

**DOI:** 10.31662/jmaj.2024-0340

**Published:** 2025-04-04

**Authors:** Takayoshi Sasako, Yann Ilboudo, Yiheng Chen, Kevin Y.H. Liang, Satoshi Yoshiji, J. Brent Richards

**Affiliations:** 1 McGill University, Montréal, Canada; 2 Lady Davis Institute for Medical Research, Jewish General Hospital, Montréal, Canada; 3 Tanaka Diabetes Clinic Omiya, Saitama, Japan; 4 Department of Diabetes and Metabolic Diseases, Graduate School of Medicine, The University of Tokyo, Tokyo, Japan; 5 Five Prime Sciences Inc, Montréal, Canada; 6 Quantitative Life Sciences Program, McGill University, Montréal, Canada; 7 The Broad Institute of MIT and Harvard, Cambridge, USA; 8 Japan Society for the Promotion of Science, Tokyo, Japan; 9 Department of Human Genetics, McGill University, Montréal, Canada; 10 Department of Epidemiology, Biostatistics and Occupational Health, McGill University, Montréal, Canada; 11 Department of Twin Research, King’s College London, London, UK

**Keywords:** androgen receptor, testosterone, trinucleotide repeat, UK Biobank, proteomics

Testosterone and its metabolites, or androgens, play pivotal roles in various tissues with a ligand-activated nuclear receptor, the androgen receptor (AR), as the key transducer protein ^(1)^. The human *AR* gene is located on chromosome X, with a CAG trinucleotide repeat encoding a polyglutamine chain and a GGC trinucleotide repeat encoding a polyglycine chain found in the first exon. A long CAG repeat and possibly a long GGC repeat are considered to suppress the AR activity as a transcription factor via conformational alteration ^(1), (2)^, and it was also suggested that they could affect the AR protein level via suppressed gene expression or protein instability ^(3), (4)^. However, it remained to be fully elucidated how the AR-mediated androgen signaling or circulating androgens could affect circulating protein levels.

Recently, we quantified CAG and GGC trinucleotide repeat lengths in the *AR* gene in nearly 200,000 European-ancestry male participants in the UK Biobank ^(5)^, in which individuals aged 40 to 69 years were enrolled between 2006 and 2010 in the United Kingdom ^(6)^. We revealed that longer CAG and GGC repeat lengths induce androgen resistance and elevate circulating total testosterone level. We also showed that total testosterone is associated with various androgen-related traits and diseases, such as fat mass, glycated hemoglobin (HbA1c), and type 2 diabetes, whereas the trinucleotide repeat lengths are associated with some of them, such as bone mineral density, male-pattern baldness, and potentially prostate cancer ^(5)^.

These results prompted us to explore circulating proteins associated with CAG or GGC trinucleotide repeat length, which are expected to serve as biomarkers to link androgen resistance and androgen-related outcomes. For that purpose, we used the Pharma Proteomics Project (PPP) data, which quantified nearly 3,000 proteins in plasma samples of over 50,000 participants in the UK Biobank using the Olink proteomics assay ^(7)^.

In this study, we identified European-ancestry male participants in the UK Biobank based on sex (data field 31, same as below) and genetic ethnic grouping (22006) ^(5)^, with Olink proteomics data from batches 1-6, not from batch 0 (pilot) or 7 (COVID-19), available. Collected baseline data include age (21022), genetic principal components (PCs; 22009), UK Biobank assessment center (54), total testosterone (30850) and sex hormone-binding globulin (SHBG; 30830) both of which were quantified by chemiluminescent immunoassay ^(5)^, fasting time (74), and body mass index (BMI; 21001). Olink proteomics data at baseline were downloaded in February 2024, and those of glioma pathogenesis-related protein 1 (GLIPR1) with low data quality ^(7)^ and SHBG also measured as stated above were excluded, leaving 2,921 proteins subject to analyses. CAG and GGC trinucleotide repeat lengths in the *AR* gene were quantified from the whole-exome sequence (WES) CRAM files using ExpansionHunter version 5.0.0 ^(8)^, as we previously reported ^(5)^. The effects of CAG and GGC repeat lengths and total testosterone which were normalized by mean and standard deviation ^(5)^, on circulating proteins which were inverse-rank normal transformed ^(9)^, were estimated by linear regression analyses. To examine the difference in betas, the Z score was calculated and converted into p value. The Bonferroni method was used for multiple-comparison correction, and the corrected p < 0.05 was considered statistically significant. All analyses were conducted and all plots were created using R Studio version 4.4.0.

CAG and GGC trinucleotide repeat lengths in the *AR* gene quantified from WES data, circulating total testosterone level, proteomics data, and other covariates were available in 14,353 male participants (Table 1). It was shown that CAG repeat length was associated with none of the proteins but GGC repeat length was slightly associated with kallikrein-related peptidase 3 (KLK3; beta, −0.04; corrected P value, 2 × 10^−3^; same as below) also known as an established tumor marker, prostate-specific antigen (PSA) ^(10)^. Moreover, total testosterone was shown to be associated positively with 136 proteins, such as insulin-like 3 (INSL3; beta, 0.24; P, 3 × 10^−113^) and prokinectin 1 (PROK1; beta, 0.23; P, 1 × 10^−99^) as well as KLK3 (beta, 0.11; P, 5 × 10^−24^), and negatively with 615 proteins, such as glucagon (GCG; beta, −0.23; P, 2 × 10^−101^), leptin (LEP; beta, −0.22; P, 2 × 10^−102^), and fatty acid binding protein 4 (FABP4; beta, −0.22; P, 5 × 10^−98^), with statistical significance (Figure 1, Supplementary Table 1).

**Table 1.** Background Characteristics of the European-Ancestry Males Subject to Regression Analyses.

Variables	Mean (SD)
Age at recruitment [year]	57.5 (8.2)
CAG trinucleotide repeat length	22.1 (3.1)
GGC trinucleotide repeat length	17.0 (2.0)
Total testosterone [nM]	12.0 (3.7)
Fasting time [hour]	3.9 (2.5)
BMI [kg/m^2^]	27.9 (4.2)

Summary of background characteristics of European-ancestry male participants in the UK Biobank for regression analyses in whom CAG and GGC trinucleotide repeat lengths in the *AR* gene quantified from whole-exome sequence data, total testosterone level, proteomics data, and other covariates were available (n = 14,353). BMI, body-mass index; SD, standard deviation.

**Figure 1.**
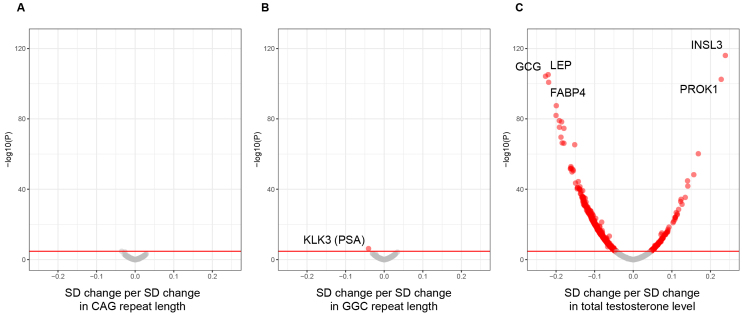
Observational associations between trinucleotide repeat lengths in the *AR* gene or total testosterone level and circulating protein levels. Linear regression analysis to estimate associations of standard deviation (SD) change in inverse rank normalized circulating protein levels per 1 SD change in CAG and GGC trinucleotide repeat lengths or total testosterone level which are independent with each other, adjusted for age, 10 ancestry PCs, assessment center, SHBG, fasting time, and batch, but not for BMI, was performed in European-ancestry male participants in the UK Biobank (n = 14,353). Volcano plots to show the betas and raw P values for associations between, (A) CAG repeat length, (B) GGC repeat length, and, (C) total testosterone level, and circulating 2,921 protein levels. CAG and GGC repeat lengths were quantified from whole-exome sequence data. P values were corrected for the 2,921 protein levels with the Bonferroni method. Associations with statistical significance (corrected p < 0.05, indicated by a red line) are indicated in red circles. AR: androgen receptor; PC: principal component; SHBG: sex hormone-binding globulin.

Given that testosterone affects body composition, especially fat mass ^(5)^, we also performed the linear regression analysis with BMI as an additional covariate. After adjustment for BMI, the association between total testosterone and LEP (beta, −0.08; P, 2 × 10^−25^) or FABP4 (beta, −0.10; P, 2 × 10^−30^) was diminished, whereas the other associations stated above were not affected. Indeed, the effect of adjustment for BMI was the largest in LEP, followed by FABP4, among all the proteins examined (Supplementary Table 1).

In this study, almost no circulating proteins were found to be specific to trinucleotide repeat lengths in the *AR* gene and serve as potential markers of androgen resistance, although GGC repeat length is slightly and negatively associated with KLK3 or PSA. We recently reported that using WES data of the UK Biobank, GGC repeat length is negatively associated with the risk of prostate cancer ^(5)^, and a previous small study reported a similar negative association despite a lack of statistical significance ^(11)^. It would be intriguing to examine whether taking GGC repeat length into account could help us detect early stages of prostate cancer or not.

LEP is an adipokine associated positively with obesity ^(7), (12)^ and negatively with androgens ^(13)^. Our study replicates the latter association and also shows that it is partially mitigated by adjustment for BMI, possibly via the negative effect of testosterone on fat mass ^(5)^. Moreover, the pro-inflammatory property of LEP is associated with diseases, and in this context, the balance with adiponectin (AdipoQ), an anti-inflammatory adipokine, and soluble leptin receptor (LEPR) to bind to and antagonize LEP are also important ^(14)^. This study shows that testosterone is not associated with AdipoQ (beta, 0.02; P, 1.0) but positively associated with LEPR with statistical significance (beta, 0.05; P, 2 × 10^−2^) (Supplementary Table 1), supporting the anti-inflammatory property of androgens ^(13)^.

Moreover, the negative association between testosterone and GCG even after adjustment for BMI shown in this study is consistent with that between total testosterone and HbA1c or type 2 diabetes ^(5)^. Androgens are known to amplify the insulinotropic action of GLP1 (glucagon-like peptide 1; not measured in the PPP) in pancreatic beta cells ^(15)^, but their roles in pancreatic alpha cells remain to be clarified, and the precise mechanisms underlying the association between testosterone and GCG should be elucidated in future studies. The positive associations between testosterone and proteins derived mainly from male reproductive tissues, such as INSL3 and PROK1, even after adjustment for BMI, could be attributed to the up-regulation of these proteins by androgens alone or in combination with insulin ^(16), (17)^.

One of the limitations of our study is that it remains to be addressed whether the identified associations are causal or not. It should be also clarified whether KLK3 level measured by Olink proteomics assay and PSA level measured in clinical practice are correlated with each other. Moreover, only cardiometabolic, inflammation, neurology, and oncology protein panels were used in the PPP ^(7)^, and other proteins were not measured.

Nevertheless, our study indicates that GGC trinucleotide repeat length in the *AR* gene is negatively associated with circulating KLK3 (PSA) level. However, circulating testosterone level is the major determinant of various circulating proteins in male participants, such as those involved in male reproduction (INSL3 and PROK1), body composition (LEP and FABP4), and metabolism (GCG). Further research is expected to reveal how androgen signaling could regulate circulating proteins and subsequently androgen-related outcomes.

## Article Information

This article is based on the study, which received the Medical Research Encouragement Prize of The Japan Medical Association in 2023.

### Conflicts of Interest

T.S. has received an endowment unrelated to this research from Eli Lilly; and personal fees unrelated to this research from Boehringer Ingelheim, Daiichi Sankyo, Eli Lilly, Kowa, Novo Nordisk, Ono, and Sumitomo. Y.C. is an employee, and J.B.R. is the founder and CEO, of 5 Prime Sciences, which provides research services for biotech, pharma, and venture capital companies for projects unrelated to this research. J.B.R. has served as an adviser to GlaxoSmithKline and Deerfield Capital. J.B.R.’s institution has received investigator-initiated grant funding from Eli Lilly, GlaxoSmithKline, and Biogen for projects unrelated to this research. The other authors have nothing to disclose.

### Sources of Funding

The J.B.R. Research Group is supported by the Canadian Institutes of Health Research (CIHR: 365825, 409511, 100558, 169303), the McGill Interdisciplinary Initiative in Infection and Immunity (MI4), the Lady Davis Institute of the Jewish General Hospital, the Jewish General Hospital Foundation, the Canadian Foundation for Innovation, the National Institute for Health Foundation, Genome Québec, the Public Health Agency of Canada, McGill University, Cancer Research UK (grant No. C18281/A29019), and the Fonds de Recherche Québec Santé (FRQS). J.B.R. is supported by an FRQS Mérite Clinical Research Scholarship. Support from Calcul Québec and Compute Canada is acknowledged. TwinsUK is funded by the Welcome Trust, Medical Research Council, European Union, the National Institute for Health Research-funded BioResource, Clinical Research Facility and Biomedical Research Centre based at Guy’s and St Thomas’ National Health Service Foundation Trust in partnership with King’s College London. T.S. is supported by the Medical Research Encouragement Prize of The Japan Medical Association and the Fund for the Promotion of Joint International Research (Fostering Joint International Research; 23KK0301) by the Japan Society for the Promotion of Science (JSPS). Y.C. has been supported by an FRQS doctoral training fellowship. S.Y. has been supported by the JSPS Overseas Research Fellowship. The aforementioned funding agencies had no role in the design, implementation, or interpretation of this study.

### Acknowledgement

We thank Dr. Tianyuan Lu (The University of Wisconsin-Madison) for giving us advice on statistical analysis.

### Author Contributions

T.S. designed this study, acquired data, performed analyses, and wrote the manuscript. Y.I. supported data acquisition and analyses. K.Y.H.L. and Y.C. supported data acquisition. S.Y. supported data analyses. J.B.R. designed this study, and reviewed and edited the manuscript. All authors contributed to the interpretation of results, critically revised the manuscript, and approved the final version. All authors agree to be accountable for all aspects of the work in ensuring that questions related to the accuracy or integrity of any part of the work are appropriately investigated and resolved. T.S. is the guarantor of this work and, as such, has full access to all the data in the study and takes responsibility for the integrity of the data and the accuracy of the data analysis.

### Approval by Institutional Review Board (IRB)

Ethics approval for the UK Biobank study was obtained from the North West Centre for Research Ethics Committee (11/NW/0382) per the Declaration of Helsinki. All participants provided informed consent at recruitment, and data of those who withdrew consent were excluded.

### Data and Code Availability

The data that support the findings of this study are available from the UK Biobank but restrictions apply to the availability of these data, which were used under license for the present study and therefore are not publicly available. Data are, however, available from the authors on reasonable request and with permission from the UK Biobank Research Committee. Computational scripts used to conduct the present study are available from the corresponding authors upon reasonable request.

## Supplement

Supplementary Table 1
