# Supplementary material for: The Influence of Trinucleotide Repeats in the Androgen Receptor Gene and Testosterone Level on Circulating Proteins in Male Participants: Proteomics Analysis Using the UK Biobank Data
Source: JMA J. 2025 Apr 4;8(2):591–5. doi: 10.31662/jmaj.2024-0340 (PMC12095775; doi:10.31662/jmaj.2024-0340)
Supplement: Supplementary Table 1 [file 2433-3298-8-2-0591-s001.pdf]

Supplementary Table 1. Observational associations between trinucleotide repeat lengths in the *AR* gene or total testosterone level and circulating protein levels with or without adjustment for BMI.

Protein	Association with proteins without adjustment for BMI				Association with proteins with adjustment for BMI				Difference in estimates with and without adjustment for BMI		
	Beta	Standard error	P value	Corrected P value	Beta	Standard error	P value	Corrected P value	Z score	P value	Corrected P value
Negatively associated with GGC repeat length											
KLK3	-0.041	0.008	5.E-07	2.E-03	-0.040	0.008	7.E-07	2.E-03	0.038	0.013	1
Positively associated with total testosterone											
INSL3	0.239	0.010	9.E-117	3.E-113	0.232	0.010	7.E-108	2.E-104	-0.432	0.177	1
PROK1	0.228	0.010	3.E-103	1.E-99	0.236	0.011	1.E-107	3.E-104	0.550	0.235	1
CA14	0.169	0.010	7.E-61	2.E-57	0.118	0.010	6.E-32	2.E-28	-3.539	3.397	1
APOD	0.157	0.011	5.E-49	2.E-45	0.099	0.010	2.E-21	6.E-18	-3.933	4.076	2.E-01
APOF	0.141	0.010	2.E-42	5.E-39	0.061	0.010	3.E-10	9.E-07	-5.691	7.898	4.E-05
IGFBP1	0.141	0.010	2.E-45	5.E-42	0.078	0.010	3.E-16	1.E-12	-4.577	5.326	1.E-02
MMP3	0.135	0.011	4.E-36	1.E-32	0.107	0.011	3.E-23	9.E-20	-1.814	1.157	1
GAL	0.126	0.011	4.E-32	1.E-28	0.087	0.011	4.E-16	1.E-12	-2.623	2.060	1
HSD11B1	0.124	0.010	6.E-35	2.E-31	0.082	0.010	2.E-16	6.E-13	-2.989	2.553	1
PON3	0.123	0.010	9.E-34	3.E-30	0.047	0.010	8.E-07	2.E-03	-5.458	7.318	1.E-04
BCAN	0.118	0.010	3.E-29	9.E-26	0.080	0.010	2.E-14	6.E-11	-2.574	1.998	1
ART3	0.114	0.011	1.E-26	3.E-23	0.080	0.011	9.E-14	2.E-10	-2.302	1.671	1
SPOCK1	0.113	0.011	4.E-26	1.E-22	0.118	0.011	2.E-27	7.E-24	0.291	0.113	1
KLK3	0.111	0.010	2.E-27	5.E-24	0.104	0.010	2.E-23	7.E-20	-0.535	0.227	1
PLA2G7	0.109	0.011	2.E-24	5.E-21	0.112	0.011	4.E-25	1.E-21	0.197	0.074	1
CA6	0.109	0.011	7.E-25	2.E-21	0.084	0.011	3.E-15	1.E-11	-1.674	1.026	1
APOM	0.108	0.011	1.E-24	3.E-21	0.067	0.010	1.E-10	4.E-07	-2.748	2.222	1
GP2	0.108	0.011	1.E-23	4.E-20	0.083	0.011	2.E-14	7.E-11	-1.658	1.012	1
NCAM1	0.105	0.011	2.E-22	5.E-19	0.081	0.011	5.E-14	2.E-10	-1.536	0.905	1
SPINK5	0.103	0.011	1.E-21	3.E-18	0.094	0.011	8.E-18	2.E-14	-0.610	0.266	1
IL17RB	0.093	0.010	3.E-19	1.E-15	0.062	0.010	2.E-09	7.E-06	-2.100	1.447	1
WFIKKN2	0.090	0.010	6.E-18	2.E-14	0.030	0.010	3.E-03	1	-4.113	4.408	1.E-01
NCAN	0.090	0.011	3.E-17	1.E-13	0.038	0.010	3.E-04	7.E-01	-3.449	3.250	1
CST6	0.089	0.011	2.E-16	6.E-13	0.070	0.011	2.E-10	5.E-07	-1.243	0.670	1
SLITRK1	0.088	0.011	2.E-16	7.E-13	0.036	0.011	6.E-04	1	-3.454	3.258	1
TNFSF10	0.087	0.011	2.E-16	7.E-13	0.103	0.011	1.E-21	3.E-18	1.055	0.536	1
LRTM2	0.085	0.011	6.E-16	2.E-12	0.040	0.010	1.E-04	4.E-01	-3.049	2.640	1
VWC2L	0.084	0.011	3.E-15	9.E-12	0.025	0.010	2.E-02	1	-3.959	4.123	2.E-01
OBP2B	0.084	0.011	6.E-15	2.E-11	0.049	0.011	6.E-06	2.E-02	-2.323	1.695	1
FGFBP1	0.082	0.011	1.E-14	3.E-11	0.049	0.011	4.E-06	1.E-02	-2.221	1.579	1
TNFSF11	0.082	0.010	5.E-15	1.E-11	0.094	0.011	4.E-19	1.E-15	0.850	0.403	1
OMG	0.079	0.010	2.E-14	6.E-11	0.066	0.010	2.E-10	7.E-07	-0.864	0.412	1
CELA2A	0.077	0.011	4.E-13	1.E-09	0.061	0.011	1.E-08	4.E-05	-1.074	0.548	1
PLA2G1B	0.077	0.011	5.E-13	1.E-09	0.042	0.011	8.E-05	2.E-01	-2.325	1.698	1
IGSF21	0.077	0.011	4.E-13	1.E-09	0.075	0.011	3.E-12	8.E-09	-0.124	0.045	1

KLK6	0.076	0.011	1.E-12	3.E-09	0.055	0.011	3.E-07	9.E-04	-1.382	0.777	1
NPTXR	0.076	0.011	1.E-12	4.E-09	0.040	0.011	2.E-04	5.E-01	-2.356	1.733	1
SYT1	0.075	0.011	1.E-12	3.E-09	0.045	0.011	2.E-05	7.E-02	-2.018	1.361	1
PTPRR	0.075	0.010	5.E-13	1.E-09	0.042	0.010	6.E-05	2.E-01	-2.272	1.637	1
HBQ1	0.074	0.010	1.E-12	4.E-09	0.072	0.011	1.E-11	3.E-08	-0.157	0.058	1
CKB	0.074	0.009	7.E-16	2.E-12	0.009	0.009	3.E-01	1	-5.159	6.605	7.E-04
NTRK3	0.074	0.011	2.E-12	7.E-09	0.024	0.010	2.E-02	1	-3.405	3.179	1
APOC1	0.073	0.011	8.E-12	2.E-08	0.049	0.011	4.E-06	1.E-02	-1.561	0.926	1
ENDOU	0.072	0.011	6.E-11	2.E-07	0.071	0.011	2.E-10	7.E-07	-0.081	0.029	1
MOG	0.072	0.010	4.E-12	1.E-08	0.033	0.010	1.E-03	1	-2.642	2.085	1
IGFBP2	0.072	0.009	6.E-15	2.E-11	0.004	0.009	6.E-01	1	-5.352	7.061	3.E-04
OPTC	0.071	0.011	3.E-11	8.E-08	0.022	0.010	3.E-02	1	-3.252	2.941	1
KLK14	0.071	0.011	4.E-11	1.E-07	0.045	0.011	3.E-05	7.E-02	-1.680	1.032	1
DNER	0.070	0.010	2.E-11	6.E-08	0.039	0.011	2.E-04	5.E-01	-2.087	1.433	1
POMC	0.070	0.011	9.E-11	3.E-07	0.088	0.011	9.E-16	3.E-12	1.162	0.611	1
MENT	0.070	0.011	7.E-11	2.E-07	0.024	0.011	2.E-02	1	-3.053	2.646	1
TNFSF12	0.069	0.011	1.E-10	3.E-07	0.056	0.011	2.E-07	5.E-04	-0.837	0.395	1
GH1	0.068	0.010	6.E-11	2.E-07	0.042	0.011	5.E-05	2.E-01	-1.750	1.096	1
MEPE	0.068	0.011	2.E-10	6.E-07	0.079	0.011	3.E-13	8.E-10	0.727	0.330	1
PODXL2	0.068	0.011	1.E-10	4.E-07	0.033	0.011	2.E-03	1	-2.322	1.694	1
GPR158	0.067	0.011	2.E-10	7.E-07	0.033	0.011	2.E-03	1	-2.257	1.620	1
APOA1	0.067	0.011	3.E-10	9.E-07	0.034	0.011	1.E-03	1	-2.168	1.520	1
SCGB1A1	0.066	0.011	3.E-10	1.E-06	0.059	0.011	4.E-08	1.E-04	-0.491	0.205	1
SUSD5	0.066	0.010	1.E-10	3.E-07	0.047	0.010	6.E-06	2.E-02	-1.319	0.727	1
GZMB	0.065	0.011	9.E-10	3.E-06	0.075	0.011	4.E-12	1.E-08	0.634	0.279	1
MSMB	0.065	0.011	1.E-09	4.E-06	0.050	0.011	3.E-06	9.E-03	-0.938	0.458	1
GHRL	0.064	0.011	2.E-09	6.E-06	0.021	0.011	5.E-02	1	-2.849	2.358	1
WIF1	0.064	0.011	1.E-09	3.E-06	0.046	0.011	1.E-05	4.E-02	-1.213	0.648	1
CRISP3	0.064	0.011	2.E-09	5.E-06	0.053	0.011	1.E-06	3.E-03	-0.755	0.347	1
TTR	0.064	0.010	8.E-10	2.E-06	0.049	0.011	3.E-06	1.E-02	-1.015	0.508	1
HS6ST2	0.064	0.011	4.E-09	1.E-05	0.060	0.011	5.E-08	2.E-04	-0.275	0.106	1
EPHA1	0.063	0.011	3.E-09	7.E-06	0.111	0.011	6.E-26	2.E-22	3.184	2.839	1
MIA	0.063	0.011	4.E-09	1.E-05	0.044	0.011	4.E-05	1.E-01	-1.239	0.667	1
SDC4	0.063	0.011	4.E-09	1.E-05	0.063	0.011	7.E-09	2.E-05	-0.007	0.002	1
ICAM4	0.063	0.011	4.E-09	1.E-05	0.053	0.011	1.E-06	3.E-03	-0.645	0.285	1
SBSN	0.063	0.011	3.E-09	1.E-05	0.069	0.011	1.E-10	4.E-07	0.422	0.172	1
WFIKKN1	0.063	0.011	4.E-09	1.E-05	0.084	0.011	4.E-15	1.E-11	1.432	0.818	1
HAGH	0.063	0.010	1.E-09	4.E-06	0.072	0.010	6.E-12	2.E-08	0.635	0.280	1
SEZ6L	0.062	0.011	5.E-09	1.E-05	0.026	0.011	1.E-02	1	-2.400	1.786	1
PAPPA	0.062	0.011	6.E-09	2.E-05	0.068	0.011	6.E-10	2.E-06	0.334	0.132	1
DSG4	0.062	0.011	4.E-09	1.E-05	0.041	0.011	9.E-05	3.E-01	-1.357	0.758	1
TEX101	0.061	0.011	2.E-08	5.E-05	0.059	0.011	5.E-08	2.E-04	-0.095	0.034	1
CD84	0.060	0.011	2.E-08	7.E-05	0.058	0.011	8.E-08	2.E-04	-0.101	0.037	1
CD38	0.059	0.011	4.E-08	1.E-04	0.092	0.011	1.E-17	3.E-14	2.184	1.538	1
DSG3	0.058	0.011	4.E-08	1.E-04	0.071	0.011	3.E-11	9.E-08	0.856	0.407	1
APOL1	0.058	0.010	2.E-08	5.E-05	0.054	0.010	3.E-07	8.E-04	-0.298	0.116	1
GFAP	0.058	0.010	1.E-08	3.E-05	0.038	0.010	2.E-04	5.E-01	-1.382	0.777	1

TPMT	0.058	0.011	7.E-08	2.E-04	0.056	0.011	3.E-07	9.E-04	-0.151	0.055	1
ENPP5	0.057	0.010	3.E-08	9.E-05	0.033	0.010	1.E-03	1	-1.656	1.010	1
RSPO1	0.057	0.010	2.E-08	5.E-05	0.064	0.010	4.E-10	1.E-06	0.497	0.208	1
VGF	0.057	0.011	6.E-08	2.E-04	0.026	0.011	2.E-02	1	-2.096	1.443	1
KIAA0319	0.057	0.011	7.E-08	2.E-04	0.021	0.011	5.E-02	1	-2.439	1.832	1
APLP1	0.056	0.011	1.E-07	4.E-04	0.019	0.011	8.E-02	1	-2.509	1.916	1
EDDM3B	0.056	0.011	1.E-07	4.E-04	0.035	0.011	1.E-03	1	-1.427	0.814	1
CCN5	0.056	0.010	9.E-09	3.E-05	0.104	0.010	8.E-28	2.E-24	3.575	3.456	1
LEG1	0.055	0.011	4.E-07	1.E-03	0.052	0.011	3.E-06	8.E-03	-0.225	0.085	1
PRDX6	0.055	0.010	1.E-07	3.E-04	0.040	0.011	1.E-04	4.E-01	-1.000	0.498	1
PON1	0.055	0.011	2.E-07	7.E-04	0.035	0.011	1.E-03	1	-1.292	0.707	1
CDH3	0.055	0.011	4.E-07	1.E-03	0.057	0.011	1.E-07	4.E-04	0.174	0.065	1
AMY2B	0.054	0.011	1.E-06	3.E-03	0.022	0.011	5.E-02	1	-2.079	1.425	1
PAEP	0.054	0.011	5.E-07	2.E-03	0.062	0.011	1.E-08	4.E-05	0.506	0.212	1
SF3B4	0.054	0.011	4.E-07	1.E-03	0.053	0.011	8.E-07	2.E-03	-0.037	0.013	1
HMBS	0.054	0.010	2.E-07	7.E-04	0.063	0.011	3.E-09	9.E-06	0.597	0.259	1
DMP1	0.054	0.010	2.E-07	7.E-04	0.024	0.010	2.E-02	1	-2.007	1.349	1
PLTP	0.054	0.010	3.E-07	9.E-04	0.011	0.010	3.E-01	1	-2.912	2.445	1
CCS	0.052	0.011	6.E-07	2.E-03	0.053	0.011	7.E-07	2.E-03	0.032	0.011	1
SCGB3A2	0.052	0.011	8.E-07	2.E-03	-0.003	0.010	8.E-01	1	-3.733	3.723	6.E-01
UMOD	0.052	0.011	9.E-07	3.E-03	0.020	0.011	6.E-02	1	-2.133	1.482	1
PTPRN2	0.052	0.011	8.E-07	2.E-03	0.026	0.011	2.E-02	1	-1.760	1.105	1
DDI2	0.052	0.010	6.E-07	2.E-03	0.053	0.011	4.E-07	1.E-03	0.092	0.033	1
PRRT3	0.052	0.011	1.E-06	4.E-03	0.016	0.011	1.E-01	1	-2.348	1.725	1
CTSV	0.051	0.010	7.E-07	2.E-03	0.052	0.010	8.E-07	2.E-03	0.024	0.008	1
CTRC	0.051	0.011	2.E-06	6.E-03	0.039	0.011	4.E-04	1	-0.821	0.385	1
CCL28	0.051	0.011	2.E-06	4.E-03	0.030	0.011	5.E-03	1	-1.388	0.782	1
PRDX2	0.050	0.010	1.E-06	3.E-03	0.057	0.010	7.E-08	2.E-04	0.421	0.172	1
CELA3A	0.050	0.011	3.E-06	8.E-03	0.037	0.011	7.E-04	1	-0.894	0.430	1
RAB6A	0.050	0.011	3.E-06	8.E-03	0.031	0.011	4.E-03	1	-1.237	0.665	1
CA2	0.050	0.010	2.E-06	5.E-03	0.060	0.011	1.E-08	4.E-05	0.674	0.301	1
MBL2	0.050	0.011	3.E-06	1.E-02	0.048	0.011	1.E-05	3.E-02	-0.116	0.042	1
CYTL1	0.049	0.011	4.E-06	1.E-02	0.026	0.011	1.E-02	1	-1.514	0.886	1
FCRL5	0.049	0.011	5.E-06	1.E-02	0.081	0.011	5.E-14	1.E-10	2.119	1.468	1
BOC	0.049	0.011	4.E-06	1.E-02	0.041	0.011	1.E-04	4.E-01	-0.527	0.223	1
SPINT3	0.049	0.010	3.E-06	9.E-03	0.026	0.011	1.E-02	1	-1.541	0.909	1
NUDT2	0.049	0.011	5.E-06	1.E-02	0.043	0.011	7.E-05	2.E-01	-0.380	0.153	1
EDAR	0.049	0.011	5.E-06	1.E-02	0.047	0.011	1.E-05	4.E-02	-0.093	0.033	1
IGDCC4	0.049	0.010	2.E-06	6.E-03	0.032	0.010	2.E-03	1	-1.127	0.585	1
IGFBP6	0.048	0.010	4.E-06	1.E-02	0.085	0.010	3.E-16	1.E-12	2.504	1.910	1
ACP1	0.048	0.011	8.E-06	2.E-02	0.046	0.011	2.E-05	7.E-02	-0.136	0.050	1
CELSR2	0.048	0.011	9.E-06	2.E-02	0.055	0.011	5.E-07	1.E-03	0.454	0.187	1
SLC4A1	0.048	0.010	5.E-06	1.E-02	0.038	0.011	4.E-04	1	-0.679	0.304	1
AMY2A	0.048	0.011	1.E-05	4.E-02	0.015	0.011	2.E-01	1	-2.121	1.469	1
APOA2	0.048	0.010	3.E-06	1.E-02	0.019	0.010	7.E-02	1	-1.988	1.329	1
LEPR	0.047	0.010	6.E-06	2.E-02	-0.011	0.010	3.E-01	1	-3.976	4.154	2.E-01
KEL	0.047	0.011	1.E-05	3.E-02	0.065	0.011	3.E-09	7.E-06	1.150	0.602	1

S100G	0.047	0.010	7.E-06	2.E-02	0.054	0.011	3.E-07	9.E-04	0.493	0.206	1
CEND1	0.047	0.011	2.E-05	5.E-02	0.030	0.011	6.E-03	1	-1.078	0.551	1
GP1BA	0.047	0.011	1.E-05	4.E-02	0.035	0.011	1.E-03	1	-0.773	0.357	1
PLB1	0.046	0.010	9.E-06	2.E-02	0.030	0.011	4.E-03	1	-1.091	0.560	1
PPME1	0.046	0.010	8.E-06	2.E-02	0.050	0.011	2.E-06	7.E-03	0.229	0.087	1
UBAC1	0.046	0.010	9.E-06	3.E-02	0.049	0.011	3.E-06	1.E-02	0.179	0.067	1
PRDX1	0.046	0.011	1.E-05	4.E-02	0.039	0.011	2.E-04	7.E-01	-0.457	0.189	1
Negatively associated with total testosterone											
GCG	-0.227	0.010	6.E-105	2.E-101	-0.202	0.010	6.E-83	2.E-79	1.691	1.041	1
LEP	-0.220	0.010	7.E-106	2.E-102	-0.080	0.007	9.E-29	2.E-25	11.472	29.737	5.E-27
FABP4	-0.219	0.010	2.E-101	5.E-98	-0.103	0.008	8.E-34	2.E-30	8.787	17.814	4.E-15
PYY	-0.200	0.010	1.E-82	3.E-79	-0.191	0.010	2.E-73	5.E-70	0.636	0.280	1
GIP	-0.199	0.010	3.E-88	1.E-84	-0.207	0.010	4.E-92	1.E-88	-0.514	0.217	1
PRAP1	-0.192	0.010	1.E-79	3.E-76	-0.118	0.010	5.E-35	1.E-31	5.295	6.924	3.E-04
PALM2	-0.191	0.010	6.E-76	2.E-72	-0.111	0.010	2.E-30	4.E-27	5.653	7.802	5.E-05
TMPRSS15	-0.187	0.011	3.E-70	8.E-67	-0.167	0.011	1.E-55	3.E-52	1.326	0.733	1
ADM	-0.186	0.010	5.E-79	1.E-75	-0.116	0.009	2.E-35	5.E-32	5.169	6.628	7.E-04
PLIN1	-0.184	0.011	5.E-67	1.E-63	-0.118	0.010	1.E-30	3.E-27	4.511	5.191	2.E-02
IL1RN	-0.180	0.010	7.E-67	2.E-63	-0.095	0.010	4.E-23	1.E-19	6.003	8.712	6.E-06
YAP1	-0.180	0.010	2.E-75	7.E-72	-0.120	0.009	3.E-37	8.E-34	4.372	4.909	4.E-02
MATN2	-0.163	0.011	3.E-52	8.E-49	-0.139	0.011	5.E-38	1.E-34	1.580	0.942	1
SEMA3F	-0.162	0.011	1.E-52	4.E-49	-0.113	0.010	3.E-27	8.E-24	3.303	3.019	1
EDN1	-0.162	0.010	1.E-53	4.E-50	-0.126	0.010	2.E-33	5.E-30	2.394	1.779	1
ADAMTSL2	-0.160	0.010	1.E-52	4.E-49	-0.085	0.010	9.E-18	3.E-14	5.198	6.695	6.E-04
TNFSF13B	-0.159	0.011	1.E-50	4.E-47	-0.131	0.011	6.E-35	2.E-31	1.821	1.163	1
IL6	-0.156	0.010	6.E-52	2.E-48	-0.101	0.010	1.E-23	3.E-20	3.875	3.971	3.E-01
TNFRSF10A	-0.155	0.010	3.E-51	9.E-48	-0.111	0.010	1.E-27	4.E-24	3.080	2.685	1
GDF15	-0.152	0.009	5.E-66	1.E-62	-0.111	0.009	2.E-37	6.E-34	3.270	2.969	1
IGSF3	-0.150	0.011	3.E-44	9.E-41	-0.074	0.010	2.E-13	7.E-10	5.117	6.507	9.E-04
ANGPTL1	-0.145	0.011	6.E-41	2.E-37	-0.148	0.011	1.E-41	4.E-38	-0.211	0.079	1
THBS2	-0.145	0.011	1.E-41	4.E-38	-0.097	0.011	6.E-20	2.E-16	3.202	2.865	1
ADAMTS15	-0.144	0.011	8.E-42	2.E-38	-0.072	0.010	1.E-12	3.E-09	4.943	6.113	2.E-03
NT5C1A	-0.142	0.010	4.E-45	1.E-41	-0.087	0.010	1.E-18	4.E-15	3.952	4.111	2.E-01
SNCG	-0.139	0.010	6.E-41	2.E-37	-0.087	0.010	2.E-17	5.E-14	3.634	3.555	8.E-01
GPD1	-0.139	0.010	8.E-41	2.E-37	-0.058	0.010	2.E-09	6.E-06	5.709	7.943	3.E-05
PPY	-0.139	0.010	2.E-40	5.E-37	-0.134	0.011	9.E-37	3.E-33	0.345	0.137	1
CFI	-0.138	0.010	4.E-42	1.E-38	-0.083	0.010	4.E-17	1.E-13	3.870	3.964	3.E-01
CSF1	-0.137	0.011	3.E-38	9.E-35	-0.092	0.010	2.E-18	7.E-15	3.049	2.640	1
MEP1A	-0.136	0.010	4.E-38	1.E-34	-0.109	0.011	8.E-25	2.E-21	1.816	1.159	1
TIMP4	-0.135	0.010	2.E-39	5.E-36	-0.112	0.010	2.E-27	6.E-24	1.581	0.944	1
MFGE8	-0.133	0.011	2.E-36	5.E-33	-0.117	0.011	8.E-28	2.E-24	1.108	0.572	1
CD4	-0.132	0.011	1.E-35	3.E-32	-0.090	0.011	1.E-17	4.E-14	2.836	2.340	1
FCAMR	-0.132	0.010	1.E-36	3.E-33	-0.072	0.010	2.E-12	5.E-09	4.162	4.500	9.E-02
ANGPT2	-0.132	0.011	5.E-36	2.E-32	-0.095	0.010	2.E-19	6.E-16	2.515	1.924	1
IGSF9	-0.131	0.010	6.E-40	2.E-36	-0.049	0.009	8.E-08	2.E-04	6.083	8.928	3.E-06
RARRES2	-0.131	0.010	2.E-36	5.E-33	-0.075	0.010	1.E-13	4.E-10	3.874	3.971	3.E-01
CD72	-0.130	0.011	4.E-34	1.E-30	-0.076	0.010	4.E-13	1.E-09	3.635	3.556	8.E-01

MYBPC1	-0.130	0.010	3.E-36	8.E-33	-0.098	0.010	3.E-21	8.E-18	2.191	1.546	1
CALB2	-0.130	0.011	3.E-33	7.E-30	-0.071	0.011	2.E-11	5.E-08	3.936	4.081	2.E-01
EPS8L2	-0.130	0.011	6.E-34	2.E-30	-0.076	0.010	3.E-13	8.E-10	3.563	3.437	1
PLA2G10	-0.129	0.010	5.E-36	1.E-32	-0.154	0.010	2.E-49	5.E-46	-1.677	1.029	1
CTSD	-0.129	0.011	4.E-32	1.E-28	-0.072	0.011	1.E-11	3.E-08	3.735	3.726	5.E-01
CDH2	-0.129	0.010	7.E-35	2.E-31	-0.068	0.010	2.E-11	5.E-08	4.192	4.559	8.E-02
FOLR2	-0.129	0.010	2.E-34	5.E-31	-0.084	0.010	5.E-16	1.E-12	3.000	2.569	1
RNASE6	-0.128	0.010	6.E-36	2.E-32	-0.077	0.010	2.E-14	6.E-11	3.576	3.457	1
CD14	-0.127	0.011	1.E-31	4.E-28	-0.129	0.011	1.E-31	3.E-28	-0.140	0.051	1
FAM3B	-0.127	0.011	4.E-32	1.E-28	-0.128	0.011	4.E-32	1.E-28	-0.114	0.041	1
GGH	-0.127	0.011	1.E-31	4.E-28	-0.072	0.011	1.E-11	3.E-08	3.608	3.511	9.E-01
FGF21	-0.126	0.010	8.E-34	2.E-30	-0.075	0.010	2.E-13	5.E-10	3.491	3.317	1
ENPP2	-0.126	0.010	6.E-33	2.E-29	-0.095	0.011	2.E-19	7.E-16	2.090	1.436	1
SCG2	-0.125	0.010	2.E-32	4.E-29	-0.133	0.011	1.E-35	3.E-32	-0.540	0.230	1
HSPB6	-0.124	0.010	1.E-35	4.E-32	-0.076	0.010	1.E-14	3.E-11	3.473	3.289	1
PDZK1	-0.124	0.011	6.E-31	2.E-27	-0.068	0.010	1.E-10	3.E-07	3.776	3.797	5.E-01
EZR	-0.124	0.011	3.E-31	1.E-27	-0.086	0.011	7.E-16	2.E-12	2.534	1.948	1
SORBS1	-0.123	0.010	7.E-32	2.E-28	-0.078	0.010	4.E-14	1.E-10	3.046	2.634	1
RBFOX3	-0.122	0.010	2.E-33	7.E-30	-0.072	0.010	4.E-13	1.E-09	3.503	3.338	1
MUC13	-0.121	0.011	6.E-29	2.E-25	-0.094	0.011	6.E-18	2.E-14	1.753	1.099	1
ALCAM	-0.121	0.011	3.E-30	8.E-27	-0.104	0.011	2.E-22	7.E-19	1.132	0.589	1
ADAM12	-0.121	0.011	5.E-30	2.E-26	-0.063	0.010	1.E-09	3.E-06	3.925	4.062	3.E-01
HGF	-0.121	0.010	5.E-31	1.E-27	-0.042	0.010	2.E-05	4.E-02	5.494	7.405	1.E-04
CD300E	-0.120	0.010	1.E-30	4.E-27	-0.069	0.010	1.E-11	4.E-08	3.475	3.292	1
BMPER	-0.119	0.011	1.E-28	4.E-25	-0.069	0.011	7.E-11	2.E-07	3.350	3.092	1
AGR3	-0.119	0.011	5.E-28	1.E-24	-0.085	0.011	3.E-15	8.E-12	2.187	1.541	1
LAIR1	-0.118	0.010	1.E-30	4.E-27	-0.064	0.010	1.E-10	4.E-07	3.746	3.746	5.E-01
GAST	-0.117	0.010	8.E-30	2.E-26	-0.109	0.010	2.E-25	5.E-22	0.548	0.234	1
TGFBR2	-0.117	0.010	2.E-29	5.E-26	-0.064	0.010	3.E-10	9.E-07	3.662	3.602	7.E-01
GOLM2	-0.117	0.011	2.E-28	5.E-25	-0.060	0.010	6.E-09	2.E-05	3.862	3.950	3.E-01
C1QTNF5	-0.117	0.011	2.E-27	7.E-24	-0.087	0.011	7.E-16	2.E-12	1.940	1.281	1
C1QTNF1	-0.116	0.011	2.E-27	5.E-24	-0.074	0.011	2.E-12	7.E-09	2.782	2.268	1
MYL3	-0.116	0.011	5.E-28	1.E-24	-0.087	0.011	2.E-16	7.E-13	1.949	1.290	1
SIGLEC7	-0.116	0.011	3.E-27	1.E-23	-0.073	0.011	6.E-12	2.E-08	2.832	2.334	1
CES1	-0.115	0.010	3.E-29	1.E-25	-0.059	0.010	4.E-09	1.E-05	3.942	4.092	2.E-01
EPO	-0.115	0.011	8.E-27	2.E-23	-0.094	0.011	4.E-18	1.E-14	1.396	0.789	1
LGALS4	-0.114	0.010	3.E-28	1.E-24	-0.076	0.010	2.E-13	7.E-10	2.645	2.087	1
SCARB2	-0.113	0.010	8.E-29	2.E-25	-0.062	0.010	6.E-10	2.E-06	3.634	3.554	8.E-01
OSMR	-0.113	0.011	3.E-26	8.E-23	-0.097	0.011	1.E-19	4.E-16	1.026	0.516	1
TNFRSF1A	-0.112	0.010	2.E-27	7.E-24	-0.057	0.010	2.E-08	5.E-05	3.823	3.880	4.E-01
CRELD1	-0.112	0.011	6.E-26	2.E-22	-0.058	0.010	3.E-08	9.E-05	3.658	3.595	7.E-01
ASGR1	-0.112	0.010	6.E-27	2.E-23	-0.047	0.010	2.E-06	7.E-03	4.502	5.171	2.E-02
TNFRSF10B	-0.110	0.010	3.E-28	8.E-25	-0.063	0.010	2.E-10	5.E-07	3.411	3.190	1
IGFBPL1	-0.110	0.010	2.E-28	6.E-25	-0.068	0.010	7.E-12	2.E-08	3.049	2.639	1
FUOM	-0.110	0.011	6.E-25	2.E-21	-0.046	0.010	9.E-06	3.E-02	4.344	4.854	4.E-02
IL16	-0.110	0.011	2.E-25	5.E-22	-0.068	0.010	7.E-11	2.E-07	2.799	2.290	1
CD79B	-0.110	0.011	2.E-25	7.E-22	-0.072	0.011	6.E-12	2.E-08	2.506	1.914	1

SEL1L	-0.110	0.011	6.E-25	2.E-21	-0.076	0.011	8.E-13	2.E-09	2.234	1.594	1
COLEC12	-0.109	0.010	2.E-27	6.E-24	-0.046	0.010	2.E-06	5.E-03	4.507	5.182	2.E-02
DTNB	-0.109	0.010	7.E-26	2.E-22	-0.069	0.010	3.E-11	9.E-08	2.778	2.262	1
LGALS9	-0.109	0.010	1.E-25	3.E-22	-0.051	0.010	4.E-07	1.E-03	3.987	4.174	2.E-01
CLMP	-0.109	0.010	5.E-26	2.E-22	-0.034	0.010	5.E-04	1	5.299	6.934	3.E-04
CD80	-0.109	0.010	3.E-25	1.E-21	-0.069	0.010	3.E-11	9.E-08	2.659	2.105	1
ALPI	-0.108	0.011	6.E-24	2.E-20	-0.104	0.011	1.E-21	4.E-18	0.291	0.113	1
CCL20	-0.108	0.011	2.E-24	7.E-21	-0.068	0.011	1.E-10	3.E-07	2.650	2.094	1
AGRN	-0.108	0.010	8.E-25	2.E-21	-0.057	0.010	3.E-08	9.E-05	3.450	3.251	1
ACTN2	-0.107	0.011	8.E-24	2.E-20	-0.089	0.011	1.E-16	4.E-13	1.195	0.634	1
CD302	-0.107	0.010	5.E-25	2.E-21	-0.071	0.010	8.E-12	2.E-08	2.485	1.888	1
ITGBL1	-0.107	0.010	1.E-26	4.E-23	-0.055	0.010	3.E-08	8.E-05	3.737	3.730	5.E-01
PLAUR	-0.107	0.010	5.E-25	1.E-21	-0.071	0.010	5.E-12	1.E-08	2.444	1.838	1
MRC1	-0.107	0.010	6.E-25	2.E-21	-0.060	0.010	5.E-09	2.E-05	3.242	2.926	1
ITGA5	-0.107	0.011	2.E-23	4.E-20	-0.041	0.010	6.E-05	2.E-01	4.422	5.010	3.E-02
GGT5	-0.107	0.011	4.E-23	1.E-19	-0.092	0.011	2.E-17	6.E-14	0.930	0.453	1
GSTA1	-0.106	0.011	9.E-24	2.E-20	-0.031	0.010	2.E-03	1	5.153	6.592	7.E-04
GHR	-0.106	0.010	7.E-27	2.E-23	-0.040	0.009	2.E-05	6.E-02	4.832	5.869	4.E-03
FGL1	-0.106	0.010	7.E-25	2.E-21	-0.121	0.010	7.E-31	2.E-27	-0.986	0.489	1
NHLRC3	-0.106	0.011	1.E-23	4.E-20	-0.058	0.010	2.E-08	6.E-05	3.217	2.887	1
SETMAR	-0.105	0.011	1.E-22	4.E-19	-0.057	0.011	9.E-08	3.E-04	3.232	2.911	1
FSTL3	-0.105	0.010	2.E-25	7.E-22	-0.043	0.010	1.E-05	3.E-02	4.453	5.072	2.E-02
LMNB2	-0.105	0.010	1.E-23	4.E-20	-0.056	0.010	5.E-08	1.E-04	3.308	3.027	1
HAVCR2	-0.105	0.010	4.E-24	1.E-20	-0.060	0.010	6.E-09	2.E-05	3.109	2.727	1
LRP11	-0.105	0.011	9.E-23	3.E-19	-0.073	0.011	1.E-11	3.E-08	2.126	1.475	1
RNASE4	-0.105	0.010	1.E-23	3.E-20	-0.054	0.010	1.E-07	4.E-04	3.492	3.320	1
NFASC	-0.105	0.010	5.E-24	1.E-20	-0.049	0.010	1.E-06	4.E-03	3.855	3.937	3.E-01
DLK1	-0.105	0.011	7.E-23	2.E-19	-0.070	0.011	5.E-11	1.E-07	2.315	1.685	1
THY1	-0.104	0.011	5.E-23	1.E-19	-0.041	0.010	7.E-05	2.E-01	4.346	4.858	4.E-02
CTSE	-0.104	0.011	6.E-22	2.E-18	-0.099	0.011	2.E-19	5.E-16	0.332	0.131	1
FABP3	-0.104	0.010	7.E-26	2.E-22	-0.050	0.010	2.E-07	6.E-04	3.898	4.014	3.E-01
GFRA1	-0.104	0.011	7.E-23	2.E-19	-0.058	0.010	3.E-08	8.E-05	3.090	2.699	1
CLSTN3	-0.103	0.011	6.E-22	2.E-18	-0.058	0.011	4.E-08	1.E-04	2.988	2.551	1
AREG	-0.103	0.010	6.E-23	2.E-19	-0.087	0.011	1.E-16	4.E-13	1.052	0.534	1
PALM	-0.103	0.010	1.E-22	3.E-19	-0.042	0.010	4.E-05	1.E-01	4.193	4.561	8.E-02
TCOF1	-0.103	0.011	3.E-22	1.E-18	-0.063	0.011	3.E-09	9.E-06	2.682	2.136	1
CD5	-0.102	0.011	1.E-21	4.E-18	-0.070	0.011	8.E-11	2.E-07	2.156	1.507	1
CD22	-0.102	0.011	1.E-21	3.E-18	-0.053	0.011	4.E-07	1.E-03	3.259	2.952	1
FLRT2	-0.102	0.011	1.E-21	4.E-18	-0.073	0.011	1.E-11	3.E-08	1.936	1.277	1
TNFRSF11A	-0.102	0.010	1.E-22	3.E-19	-0.047	0.010	4.E-06	1.E-02	3.782	3.808	5.E-01
CHI3L1	-0.102	0.010	1.E-22	4.E-19	-0.073	0.010	3.E-12	8.E-09	1.955	1.295	1
CGREF1	-0.101	0.011	4.E-21	1.E-17	-0.072	0.011	2.E-11	7.E-08	1.916	1.256	1
PAMR1	-0.101	0.010	4.E-22	1.E-18	-0.044	0.010	2.E-05	4.E-02	3.908	4.031	3.E-01
MSR1	-0.101	0.010	3.E-23	9.E-20	-0.051	0.010	4.E-07	1.E-03	3.532	3.385	1
LGALS3BP	-0.101	0.011	1.E-21	3.E-18	-0.034	0.010	9.E-04	1	4.606	5.387	1.E-02
CFH	-0.101	0.011	1.E-21	4.E-18	-0.022	0.010	3.E-02	1	5.476	7.361	1.E-04
HS6ST1	-0.100	0.011	5.E-21	2.E-17	-0.078	0.011	4.E-13	1.E-09	1.462	0.842	1

CD300A	-0.100	0.011	1.E-20	3.E-17	-0.062	0.011	7.E-09	2.E-05	2.535	1.948	1
CBLN4	-0.100	0.011	4.E-21	1.E-17	-0.064	0.011	1.E-09	4.E-06	2.378	1.759	1
NPC2	-0.100	0.010	1.E-22	4.E-19	-0.048	0.010	1.E-06	4.E-03	3.617	3.526	9.E-01
ADAMTSL4	-0.100	0.011	2.E-20	4.E-17	-0.095	0.011	2.E-18	7.E-15	0.306	0.120	1
SMOC1	-0.100	0.010	9.E-22	3.E-18	-0.061	0.010	4.E-09	1.E-05	2.629	2.068	1
IL12B	-0.100	0.011	6.E-21	2.E-17	-0.060	0.011	1.E-08	4.E-05	2.662	2.109	1
PHOSPHO1	-0.100	0.011	4.E-21	1.E-17	-0.061	0.011	8.E-09	2.E-05	2.608	2.041	1
SLITRK2	-0.099	0.011	2.E-20	5.E-17	-0.051	0.011	1.E-06	4.E-03	3.193	2.851	1
CFB	-0.099	0.010	2.E-21	7.E-18	-0.046	0.010	7.E-06	2.E-02	3.617	3.525	9.E-01
GUSB	-0.098	0.011	4.E-20	1.E-16	-0.029	0.010	5.E-03	1	4.695	5.573	8.E-03
BCL2	-0.097	0.011	3.E-20	9.E-17	-0.068	0.011	2.E-10	5.E-07	1.983	1.324	1
LAMP2	-0.097	0.010	2.E-20	6.E-17	-0.055	0.010	1.E-07	3.E-04	2.844	2.351	1
IL12A_IL12B	-0.097	0.011	1.E-19	3.E-16	-0.058	0.011	5.E-08	1.E-04	2.589	2.017	1
CD276	-0.097	0.010	4.E-21	1.E-17	-0.077	0.010	1.E-13	3.E-10	1.356	0.757	1
ROBO2	-0.097	0.011	1.E-19	3.E-16	-0.075	0.011	4.E-12	1.E-08	1.482	0.859	1
EFEMP1	-0.097	0.010	4.E-24	1.E-20	-0.056	0.009	3.E-09	1.E-05	3.055	2.648	1
GZMA	-0.096	0.011	2.E-19	5.E-16	-0.056	0.011	1.E-07	4.E-04	2.701	2.161	1
C3	-0.096	0.011	1.E-19	3.E-16	-0.041	0.010	8.E-05	2.E-01	3.726	3.711	6.E-01
CD300C	-0.096	0.011	3.E-19	1.E-15	-0.057	0.011	9.E-08	3.E-04	2.570	1.993	1
CKAP4	-0.095	0.010	4.E-20	1.E-16	-0.056	0.010	5.E-08	2.E-04	2.684	2.138	1
VWA1	-0.095	0.011	3.E-19	8.E-16	-0.061	0.011	1.E-08	3.E-05	2.303	1.671	1
CHCHD10	-0.095	0.010	6.E-20	2.E-16	-0.046	0.010	8.E-06	2.E-02	3.372	3.127	1
IGFBP7	-0.095	0.010	9.E-20	3.E-16	-0.051	0.010	8.E-07	2.E-03	2.999	2.567	1
DPT	-0.095	0.010	4.E-20	1.E-16	-0.022	0.010	3.E-02	1	5.147	6.578	8.E-04
ASAH2	-0.094	0.011	1.E-18	3.E-15	-0.046	0.011	1.E-05	4.E-02	3.247	2.934	1
FGF19	-0.094	0.011	5.E-19	2.E-15	-0.116	0.011	2.E-27	7.E-24	-1.437	0.822	1
S100P	-0.094	0.011	1.E-18	4.E-15	-0.081	0.011	9.E-14	3.E-10	0.887	0.426	1
FAM3D	-0.094	0.011	7.E-19	2.E-15	-0.091	0.011	3.E-17	8.E-14	0.212	0.080	1
CTSZ	-0.094	0.011	1.E-18	4.E-15	-0.050	0.011	2.E-06	6.E-03	2.920	2.456	1
FURIN	-0.094	0.010	1.E-19	3.E-16	-0.026	0.010	9.E-03	1	4.759	5.710	6.E-03
OCLN	-0.094	0.010	2.E-19	7.E-16	-0.054	0.010	2.E-07	6.E-04	2.720	2.185	1
TNFRSF12A	-0.094	0.010	3.E-19	9.E-16	-0.059	0.010	2.E-08	6.E-05	2.375	1.755	1
C2	-0.093	0.011	2.E-18	6.E-15	-0.061	0.011	1.E-08	4.E-05	2.169	1.522	1
DSC2	-0.093	0.010	5.E-19	1.E-15	-0.072	0.011	8.E-12	2.E-08	1.433	0.819	1
NOS3	-0.093	0.011	6.E-18	2.E-14	-0.046	0.011	2.E-05	6.E-02	3.137	2.768	1
AGR2	-0.093	0.011	1.E-17	3.E-14	-0.100	0.011	2.E-19	4.E-16	-0.432	0.177	1
SCARA5	-0.093	0.010	4.E-19	1.E-15	-0.042	0.010	5.E-05	1.E-01	3.521	3.366	1
CCL15	-0.093	0.010	1.E-18	3.E-15	-0.063	0.011	3.E-09	8.E-06	2.030	1.373	1
RNASE1	-0.093	0.010	3.E-20	9.E-17	-0.035	0.010	3.E-04	9.E-01	4.102	4.387	1.E-01
CLEC14A	-0.092	0.011	3.E-18	1.E-14	-0.078	0.011	3.E-13	9.E-10	0.934	0.456	1
NTRK2	-0.092	0.011	1.E-17	3.E-14	-0.054	0.011	5.E-07	1.E-03	2.497	1.902	1
TFPI2	-0.092	0.011	1.E-17	3.E-14	-0.072	0.011	2.E-11	6.E-08	1.267	0.688	1
PTS	-0.092	0.011	1.E-17	4.E-14	-0.032	0.010	2.E-03	1	3.963	4.130	2.E-01
FGF23	-0.092	0.011	5.E-18	1.E-14	-0.052	0.011	1.E-06	3.E-03	2.690	2.146	1
PCBD1	-0.092	0.011	6.E-18	2.E-14	-0.028	0.010	6.E-03	1	4.323	4.813	4.E-02
CXCL16	-0.092	0.011	6.E-18	2.E-14	-0.067	0.011	4.E-10	1.E-06	1.645	1.000	1
CEACAM20	-0.092	0.011	1.E-17	4.E-14	-0.087	0.011	1.E-15	4.E-12	0.307	0.120	1

NOS1	-0.091	0.011	1.E-17	4.E-14	-0.062	0.011	9.E-09	3.E-05	1.948	1.289	1
GFRAL	-0.091	0.011	1.E-17	3.E-14	-0.073	0.011	1.E-11	3.E-08	1.195	0.634	1
FCER2	-0.091	0.011	2.E-17	6.E-14	-0.051	0.011	2.E-06	7.E-03	2.695	2.152	1
PRL	-0.091	0.011	1.E-17	4.E-14	-0.080	0.011	1.E-13	3.E-10	0.700	0.315	1
ADH4	-0.091	0.011	8.E-18	2.E-14	-0.022	0.010	3.E-02	1	4.749	5.690	6.E-03
COL5A1	-0.091	0.011	3.E-17	9.E-14	-0.063	0.011	5.E-09	2.E-05	1.814	1.157	1
CDA	-0.091	0.011	6.E-18	2.E-14	-0.061	0.011	7.E-09	2.E-05	1.983	1.324	1
SPRR3	-0.090	0.011	2.E-17	7.E-14	-0.087	0.011	8.E-16	2.E-12	0.208	0.078	1
CPM	-0.090	0.010	5.E-18	1.E-14	-0.014	0.010	2.E-01	1	5.316	6.975	3.E-04
LSP1	-0.090	0.010	7.E-18	2.E-14	-0.056	0.010	8.E-08	2.E-04	2.295	1.663	1
CFC1	-0.090	0.011	5.E-17	2.E-13	-0.047	0.011	1.E-05	3.E-02	2.850	2.359	1
IL1RL2	-0.090	0.011	8.E-17	2.E-13	-0.081	0.011	1.E-13	3.E-10	0.567	0.244	1
EPHA2	-0.090	0.010	1.E-17	3.E-14	-0.063	0.011	3.E-09	7.E-06	1.820	1.163	1
CSF1R	-0.089	0.011	6.E-17	2.E-13	-0.043	0.011	5.E-05	1.E-01	3.087	2.695	1
SIT1	-0.089	0.011	5.E-17	2.E-13	-0.057	0.011	9.E-08	3.E-04	2.137	1.486	1
RNASET2	-0.089	0.011	5.E-17	2.E-13	-0.053	0.011	6.E-07	2.E-03	2.404	1.790	1
PRND	-0.088	0.011	8.E-17	2.E-13	-0.074	0.011	6.E-12	2.E-08	0.968	0.478	1
ENAH	-0.088	0.011	2.E-16	6.E-13	-0.055	0.011	3.E-07	9.E-04	2.192	1.547	1
CDHR2	-0.088	0.011	1.E-16	3.E-13	-0.009	0.010	4.E-01	1	5.458	7.317	1.E-04
ACY1	-0.088	0.010	5.E-17	2.E-13	-0.017	0.010	8.E-02	1	4.874	5.960	3.E-03
AFM	-0.088	0.010	2.E-17	6.E-14	-0.012	0.010	2.E-01	1	5.318	6.979	3.E-04
PZP	-0.088	0.011	1.E-16	3.E-13	-0.070	0.011	7.E-11	2.E-07	1.212	0.647	1
UPB1	-0.088	0.010	3.E-17	8.E-14	-0.021	0.010	4.E-02	1	4.655	5.489	9.E-03
CD28	-0.088	0.011	7.E-16	2.E-12	-0.054	0.011	5.E-07	2.E-03	2.163	1.516	1
LBP	-0.087	0.011	3.E-16	8.E-13	-0.056	0.011	2.E-07	6.E-04	2.113	1.460	1
LECT2	-0.087	0.010	1.E-19	4.E-16	-0.052	0.010	5.E-08	2.E-04	2.560	1.980	1
LRRC25	-0.087	0.011	6.E-16	2.E-12	-0.054	0.011	5.E-07	2.E-03	2.166	1.518	1
CD74	-0.087	0.010	4.E-17	1.E-13	-0.044	0.010	2.E-05	5.E-02	2.953	2.502	1
THBS4	-0.087	0.011	3.E-16	9.E-13	-0.025	0.010	1.E-02	1	4.155	4.489	9.E-02
ACTA2	-0.087	0.010	2.E-19	5.E-16	-0.078	0.010	1.E-15	3.E-12	0.643	0.284	1
CLSTN2	-0.086	0.011	3.E-16	1.E-12	-0.064	0.011	2.E-09	5.E-06	1.467	0.846	1
VSTM2L	-0.086	0.011	1.E-15	3.E-12	-0.060	0.011	3.E-08	9.E-05	1.736	1.083	1
VSIG4	-0.086	0.010	2.E-17	7.E-14	-0.035	0.010	4.E-04	1	3.564	3.437	1
STC1	-0.086	0.011	5.E-16	1.E-12	-0.034	0.010	9.E-04	1	3.470	3.284	1
MAD1L1	-0.086	0.011	5.E-16	1.E-12	-0.043	0.010	4.E-05	1.E-01	2.880	2.400	1
AMBP	-0.086	0.010	2.E-16	5.E-13	-0.053	0.010	4.E-07	1.E-03	2.232	1.592	1
CD7	-0.085	0.011	4.E-15	1.E-11	-0.068	0.011	5.E-10	2.E-06	1.122	0.582	1
CALCA	-0.085	0.011	3.E-15	9.E-12	-0.048	0.011	7.E-06	2.E-02	2.407	1.793	1
AGRP	-0.085	0.010	3.E-16	8.E-13	-0.076	0.010	3.E-13	9.E-10	0.555	0.237	1
GRN	-0.085	0.011	5.E-15	1.E-11	-0.053	0.011	1.E-06	3.E-03	2.088	1.435	1
ADGRG1	-0.084	0.010	5.E-17	2.E-13	-0.018	0.010	7.E-02	1	4.810	5.822	4.E-03
CD48	-0.084	0.011	5.E-15	1.E-11	-0.061	0.011	2.E-08	6.E-05	1.532	0.901	1
IL18R1	-0.084	0.011	2.E-15	5.E-12	-0.030	0.010	3.E-03	1	3.646	3.575	8.E-01
FST	-0.084	0.011	2.E-15	7.E-12	-0.074	0.011	5.E-12	1.E-08	0.639	0.281	1
ADH1B	-0.084	0.011	3.E-15	8.E-12	-0.020	0.010	5.E-02	1	4.344	4.854	4.E-02
ROBO1	-0.084	0.011	6.E-15	2.E-11	-0.039	0.011	2.E-04	7.E-01	2.957	2.508	1
BAG3	-0.084	0.011	8.E-15	2.E-11	-0.042	0.011	8.E-05	2.E-01	2.733	2.203	1

POF1B	-0.083	0.011	8.E-15	2.E-11	-0.082	0.011	4.E-14	1.E-10	0.070	0.025	1
GPR37	-0.083	0.011	6.E-15	2.E-11	-0.061	0.011	2.E-08	5.E-05	1.484	0.861	1
BPIFB2	-0.083	0.010	2.E-15	6.E-12	-0.018	0.010	7.E-02	1	4.446	5.058	3.E-02
FGA	-0.083	0.010	2.E-15	6.E-12	-0.045	0.010	2.E-05	5.E-02	2.582	2.008	1
FAM20A	-0.083	0.010	1.E-15	3.E-12	-0.037	0.010	4.E-04	1	3.200	2.863	1
HEPH	-0.083	0.011	3.E-15	9.E-12	-0.036	0.010	5.E-04	1	3.165	2.809	1
CHCHD6	-0.083	0.011	5.E-15	1.E-11	-0.053	0.011	7.E-07	2.E-03	2.011	1.353	1
BAIAP2	-0.083	0.011	1.E-14	3.E-11	-0.035	0.011	1.E-03	1	3.189	2.845	1
IL10RB	-0.083	0.011	5.E-15	2.E-11	-0.038	0.010	3.E-04	8.E-01	2.990	2.555	1
VSIG2	-0.083	0.010	1.E-15	4.E-12	-0.071	0.010	1.E-11	4.E-08	0.806	0.376	1
FETUB	-0.083	0.011	7.E-15	2.E-11	-0.052	0.011	1.E-06	3.E-03	2.042	1.386	1
PAM	-0.083	0.011	1.E-14	3.E-11	-0.057	0.011	1.E-07	4.E-04	1.713	1.062	1
CEACAM16	-0.082	0.011	8.E-15	2.E-11	-0.063	0.011	4.E-09	1.E-05	1.281	0.699	1
HNMT	-0.082	0.011	1.E-14	3.E-11	-0.019	0.010	6.E-02	1	4.247	4.664	6.E-02
ELN	-0.082	0.008	5.E-22	1.E-18	-0.059	0.009	3.E-12	9.E-09	1.861	1.203	1
GPRC5C	-0.082	0.011	1.E-14	4.E-11	-0.055	0.011	2.E-07	6.E-04	1.753	1.099	1
CPXM2	-0.082	0.010	5.E-15	1.E-11	-0.035	0.010	6.E-04	1	3.174	2.823	1
CXCL13	-0.081	0.011	1.E-14	4.E-11	-0.052	0.011	9.E-07	3.E-03	1.947	1.288	1
ENO3	-0.081	0.010	9.E-15	3.E-11	-0.039	0.010	2.E-04	6.E-01	2.884	2.407	1
DRAXIN	-0.081	0.011	4.E-14	1.E-10	-0.062	0.011	1.E-08	3.E-05	1.271	0.691	1
COL3A1	-0.081	0.011	3.E-14	1.E-10	-0.054	0.011	4.E-07	1.E-03	1.746	1.092	1
SCARF2	-0.081	0.010	3.E-17	9.E-14	-0.081	0.010	9.E-17	3.E-13	0.011	0.004	1
SHISA5	-0.081	0.010	7.E-16	2.E-12	-0.036	0.010	3.E-04	9.E-01	3.207	2.873	1
TNFRSF1B	-0.081	0.010	1.E-14	4.E-11	-0.036	0.010	5.E-04	1	3.040	2.626	1
TNFRSF6B	-0.080	0.011	4.E-14	1.E-10	-0.049	0.011	5.E-06	1.E-02	2.114	1.461	1
TIMP1	-0.080	0.011	5.E-14	2.E-10	-0.039	0.011	2.E-04	6.E-01	2.735	2.205	1
AHNAK	-0.080	0.011	6.E-14	2.E-10	-0.023	0.010	2.E-02	1	3.809	3.855	4.E-01
CD163	-0.080	0.011	8.E-14	2.E-10	-0.024	0.010	2.E-02	1	3.724	3.708	6.E-01
SERPINF1	-0.080	0.010	8.E-15	2.E-11	-0.014	0.010	1.E-01	1	4.592	5.357	1.E-02
PTGR1	-0.080	0.010	3.E-14	8.E-11	-0.023	0.010	3.E-02	1	3.884	3.988	3.E-01
LAYN	-0.080	0.010	6.E-15	2.E-11	-0.072	0.010	4.E-12	1.E-08	0.541	0.230	1
PVR	-0.080	0.011	8.E-14	2.E-10	-0.062	0.011	1.E-08	3.E-05	1.200	0.638	1
HAVCR1	-0.080	0.010	5.E-16	1.E-12	-0.036	0.010	2.E-04	6.E-01	3.166	2.810	1
DCTPP1	-0.080	0.011	6.E-14	2.E-10	-0.059	0.011	3.E-08	9.E-05	1.351	0.753	1
ULBP2	-0.080	0.011	9.E-14	3.E-10	-0.050	0.011	3.E-06	8.E-03	1.945	1.285	1
KRT18	-0.080	0.011	1.E-13	3.E-10	-0.004	0.010	7.E-01	1	5.107	6.485	1.E-03
LILRA2	-0.079	0.011	2.E-13	5.E-10	-0.038	0.011	3.E-04	1	2.692	2.149	1
PLXNB2	-0.079	0.011	7.E-14	2.E-10	-0.037	0.010	4.E-04	1	2.827	2.329	1
CEMIP2	-0.079	0.011	3.E-13	8.E-10	-0.045	0.011	2.E-05	7.E-02	2.183	1.537	1
FBP1	-0.079	0.011	3.E-13	7.E-10	-0.039	0.011	3.E-04	9.E-01	2.643	2.085	1
ANGPTL4	-0.079	0.010	7.E-14	2.E-10	-0.030	0.010	3.E-03	1	3.274	2.974	1
FCRL1	-0.078	0.011	4.E-13	1.E-09	-0.043	0.011	7.E-05	2.E-01	2.325	1.698	1
B2M	-0.078	0.010	3.E-14	7.E-11	-0.042	0.010	4.E-05	1.E-01	2.476	1.877	1
NADK	-0.078	0.010	6.E-14	2.E-10	-0.049	0.010	3.E-06	9.E-03	2.001	1.343	1
CRIP2	-0.078	0.010	9.E-14	3.E-10	-0.034	0.010	9.E-04	1	2.961	2.513	1
RBP5	-0.078	0.010	1.E-13	4.E-10	-0.005	0.010	6.E-01	1	5.049	6.353	1.E-03
HIP1R	-0.078	0.011	2.E-13	7.E-10	-0.034	0.011	1.E-03	1	2.944	2.490	1

IMMT	-0.077	0.011	3.E-13	7.E-10	-0.034	0.010	1.E-03	1	2.924	2.461	1
IL15	-0.077	0.011	6.E-13	2.E-09	-0.077	0.011	1.E-12	4.E-09	0.000	0.000	1
AXL	-0.077	0.011	6.E-13	2.E-09	-0.053	0.011	9.E-07	3.E-03	1.590	0.952	1
PXN	-0.077	0.011	5.E-13	1.E-09	-0.046	0.011	2.E-05	5.E-02	2.050	1.394	1
LTA	-0.077	0.011	9.E-13	3.E-09	-0.050	0.011	4.E-06	1.E-02	1.754	1.100	1
PTH	-0.077	0.011	4.E-13	1.E-09	-0.048	0.011	7.E-06	2.E-02	1.945	1.285	1
GGT1	-0.077	0.011	7.E-13	2.E-09	-0.017	0.010	1.E-01	1	4.000	4.199	2.E-01
SPON1	-0.077	0.010	2.E-13	6.E-10	-0.056	0.010	1.E-07	4.E-04	1.416	0.805	1
MILR1	-0.076	0.011	1.E-12	4.E-09	-0.053	0.011	8.E-07	2.E-03	1.492	0.868	1
IDUA	-0.076	0.011	1.E-12	4.E-09	-0.038	0.011	4.E-04	1	2.505	1.912	1
LILRB1	-0.076	0.011	1.E-12	4.E-09	-0.044	0.011	5.E-05	1.E-01	2.140	1.490	1
ICAM1	-0.076	0.011	2.E-12	5.E-09	-0.038	0.011	4.E-04	1	2.515	1.925	1
GIPC2	-0.076	0.010	2.E-13	7.E-10	-0.052	0.010	7.E-07	2.E-03	1.634	0.991	1
DLL1	-0.076	0.011	1.E-12	3.E-09	-0.048	0.011	7.E-06	2.E-02	1.833	1.175	1
B4GALT1	-0.076	0.011	1.E-12	3.E-09	-0.050	0.011	3.E-06	9.E-03	1.701	1.051	1
FOLR3	-0.075	0.011	2.E-12	5.E-09	-0.046	0.011	2.E-05	5.E-02	1.918	1.259	1
CCN1	-0.075	0.011	1.E-12	4.E-09	-0.060	0.011	3.E-08	8.E-05	1.044	0.528	1
RNF149	-0.075	0.011	1.E-12	4.E-09	-0.049	0.011	5.E-06	1.E-02	1.748	1.095	1
LYVE1	-0.075	0.011	2.E-12	6.E-09	-0.053	0.011	8.E-07	2.E-03	1.450	0.832	1
VNN2	-0.075	0.011	3.E-12	9.E-09	-0.055	0.011	3.E-07	1.E-03	1.279	0.697	1
ADAM8	-0.075	0.011	3.E-12	8.E-09	-0.044	0.011	4.E-05	1.E-01	2.028	1.371	1
LILRB4	-0.075	0.010	8.E-13	2.E-09	-0.030	0.010	4.E-03	1	3.061	2.657	1
FTCD	-0.075	0.010	9.E-13	3.E-09	-0.005	0.010	7.E-01	1	4.847	5.903	4.E-03
ACY3	-0.075	0.011	5.E-12	2.E-08	-0.043	0.011	9.E-05	3.E-01	2.095	1.441	1
SKAP1	-0.074	0.011	2.E-12	7.E-09	-0.048	0.011	7.E-06	2.E-02	1.764	1.109	1
DPY30	-0.074	0.011	2.E-12	6.E-09	-0.036	0.011	7.E-04	1	2.588	2.015	1
DPP4	-0.074	0.011	3.E-12	1.E-08	-0.060	0.011	2.E-08	6.E-05	0.914	0.443	1
LIFR	-0.074	0.011	5.E-12	1.E-08	-0.052	0.011	2.E-06	5.E-03	1.464	0.844	1
GSTA3	-0.074	0.011	5.E-12	2.E-08	-0.009	0.010	4.E-01	1	4.369	4.903	4.E-02
SIGLEC6	-0.074	0.011	6.E-12	2.E-08	-0.040	0.011	2.E-04	5.E-01	2.208	1.564	1
BST1	-0.074	0.011	7.E-12	2.E-08	-0.062	0.011	1.E-08	3.E-05	0.744	0.340	1
KRT8	-0.073	0.011	1.E-11	3.E-08	-0.039	0.011	4.E-04	1	2.282	1.648	1
HSD17B14	-0.073	0.011	7.E-12	2.E-08	-0.061	0.011	1.E-08	4.E-05	0.769	0.355	1
PLA2G2A	-0.073	0.011	5.E-12	1.E-08	-0.047	0.011	1.E-05	3.E-02	1.757	1.102	1
CST3	-0.073	0.010	2.E-12	5.E-09	-0.023	0.010	3.E-02	1	3.481	3.302	1
IFI30	-0.073	0.010	1.E-12	4.E-09	-0.026	0.010	1.E-02	1	3.259	2.952	1
NPDC1	-0.073	0.010	4.E-12	1.E-08	-0.048	0.011	6.E-06	2.E-02	1.678	1.029	1
F9	-0.072	0.010	3.E-12	1.E-08	-0.024	0.010	2.E-02	1	3.295	3.007	1
CR1	-0.072	0.011	1.E-11	3.E-08	-0.027	0.011	1.E-02	1	3.052	2.643	1
C1RL	-0.072	0.011	7.E-12	2.E-08	-0.035	0.011	9.E-04	1	2.502	1.908	1
BST2	-0.072	0.011	1.E-11	4.E-08	-0.033	0.011	2.E-03	1	2.571	1.994	1
GSR	-0.072	0.011	1.E-11	4.E-08	-0.041	0.011	1.E-04	3.E-01	2.049	1.393	1
TFF1	-0.072	0.010	3.E-12	9.E-09	-0.059	0.010	2.E-08	5.E-05	0.883	0.423	1
CLEC4G	-0.072	0.011	2.E-11	5.E-08	-0.047	0.011	1.E-05	4.E-02	1.658	1.012	1
CHRDL1	-0.072	0.010	4.E-13	1.E-09	-0.038	0.010	1.E-04	3.E-01	2.388	1.771	1
GRPEL1	-0.071	0.011	3.E-11	1.E-07	-0.045	0.011	3.E-05	1.E-01	1.741	1.088	1
LILRA5	-0.071	0.011	3.E-11	8.E-08	-0.024	0.011	2.E-02	1	3.140	2.773	1

PHLDB1	-0.071	0.010	8.E-12	2.E-08	-0.036	0.010	6.E-04	1	2.400	1.785	1
VEGFA	-0.071	0.011	2.E-11	6.E-08	-0.042	0.011	8.E-05	2.E-01	1.933	1.273	1
MAMDC2	-0.071	0.010	7.E-12	2.E-08	-0.041	0.010	1.E-04	3.E-01	2.085	1.431	1
ST6GAL1	-0.071	0.011	2.E-11	6.E-08	-0.041	0.011	1.E-04	4.E-01	2.036	1.380	1
ADAM9	-0.071	0.011	2.E-11	4.E-08	-0.038	0.011	3.E-04	9.E-01	2.211	1.568	1
EFNA4	-0.071	0.011	2.E-11	5.E-08	-0.022	0.010	3.E-02	1	3.322	3.050	1
LAMB1	-0.071	0.011	5.E-11	1.E-07	-0.042	0.011	1.E-04	4.E-01	1.926	1.267	1
SERPIND1	-0.071	0.010	8.E-12	2.E-08	-0.036	0.010	5.E-04	1	2.395	1.779	1
LCP1	-0.071	0.011	3.E-11	9.E-08	-0.044	0.011	4.E-05	1.E-01	1.779	1.123	1
GUCA2A	-0.071	0.011	3.E-11	8.E-08	-0.090	0.011	3.E-17	1.E-13	-1.315	0.725	1
CBS	-0.070	0.011	6.E-11	2.E-07	-0.027	0.011	1.E-02	1	2.843	2.350	1
TNF	-0.070	0.011	5.E-11	1.E-07	-0.037	0.011	6.E-04	1	2.232	1.591	1
IL6ST	-0.070	0.011	8.E-11	2.E-07	-0.052	0.011	2.E-06	6.E-03	1.196	0.635	1
KCTD5	-0.070	0.011	5.E-11	1.E-07	-0.027	0.011	1.E-02	1	2.873	2.391	1
CCL7	-0.070	0.010	3.E-11	8.E-08	-0.026	0.010	1.E-02	1	2.955	2.505	1
SOD3	-0.070	0.010	2.E-11	6.E-08	-0.073	0.011	5.E-12	1.E-08	-0.218	0.082	1
JAM2	-0.069	0.011	4.E-11	1.E-07	-0.041	0.011	1.E-04	3.E-01	1.923	1.263	1
CDCP1	-0.069	0.010	4.E-13	1.E-09	-0.045	0.010	3.E-06	9.E-03	1.804	1.147	1
MMP12	-0.069	0.010	2.E-12	6.E-09	-0.057	0.010	1.E-08	3.E-05	0.873	0.417	1
ANGPTL3	-0.069	0.011	1.E-10	3.E-07	-0.062	0.011	1.E-08	3.E-05	0.478	0.199	1
PLA2G15	-0.069	0.011	9.E-11	3.E-07	-0.018	0.011	8.E-02	1	3.395	3.163	1
FMNL1	-0.069	0.011	1.E-10	3.E-07	-0.041	0.011	2.E-04	5.E-01	1.873	1.214	1
HEG1	-0.069	0.011	2.E-10	5.E-07	-0.029	0.011	7.E-03	1	2.623	2.060	1
ISM1	-0.069	0.011	2.E-10	5.E-07	-0.022	0.011	4.E-02	1	3.077	2.680	1
SERPINA7	-0.069	0.011	1.E-10	4.E-07	-0.039	0.011	3.E-04	9.E-01	1.976	1.317	1
FBLN2	-0.068	0.010	8.E-11	2.E-07	-0.022	0.010	3.E-02	1	3.129	2.757	1
MME	-0.068	0.011	2.E-10	5.E-07	-0.025	0.011	2.E-02	1	2.882	2.403	1
MEP1B	-0.068	0.011	2.E-10	7.E-07	-0.035	0.011	1.E-03	1	2.136	1.486	1
ACE2	-0.068	0.011	2.E-10	7.E-07	-0.013	0.010	2.E-01	1	3.688	3.645	7.E-01
TFF2	-0.068	0.010	6.E-11	2.E-07	-0.056	0.010	1.E-07	3.E-04	0.806	0.376	1
DHPS	-0.068	0.011	2.E-10	5.E-07	-0.038	0.011	3.E-04	9.E-01	1.951	1.292	1
ENPP7	-0.068	0.011	6.E-10	2.E-06	-0.028	0.011	1.E-02	1	2.583	2.009	1
CTHRC1	-0.068	0.010	5.E-11	1.E-07	-0.009	0.010	4.E-01	1	4.114	4.410	1.E-01
IL10	-0.067	0.011	5.E-10	1.E-06	-0.066	0.011	2.E-09	6.E-06	0.106	0.038	1
MEGF10	-0.067	0.011	2.E-10	7.E-07	-0.074	0.011	5.E-12	1.E-08	-0.472	0.196	1
CR2	-0.067	0.011	2.E-10	6.E-07	-0.039	0.011	2.E-04	6.E-01	1.858	1.200	1
AGXT	-0.067	0.011	3.E-10	1.E-06	-0.026	0.011	2.E-02	1	2.762	2.241	1
HS3ST3B1	-0.067	0.011	5.E-10	1.E-06	-0.068	0.011	5.E-10	2.E-06	-0.051	0.018	1
IL12RB1	-0.067	0.011	5.E-10	1.E-06	-0.039	0.011	3.E-04	9.E-01	1.829	1.171	1
SIGLEC9	-0.067	0.011	5.E-10	1.E-06	-0.047	0.011	1.E-05	4.E-02	1.294	0.709	1
FSTL1	-0.067	0.011	4.E-10	1.E-06	-0.022	0.011	3.E-02	1	2.952	2.501	1
VMO1	-0.067	0.011	4.E-10	1.E-06	-0.047	0.011	2.E-05	5.E-02	1.333	0.738	1
CD93	-0.067	0.011	7.E-10	2.E-06	-0.063	0.011	9.E-09	3.E-05	0.243	0.092	1
AHNAK2	-0.067	0.011	6.E-10	2.E-06	-0.038	0.011	4.E-04	1	1.872	1.213	1
TNFRSF11B	-0.067	0.010	1.E-11	4.E-08	-0.041	0.010	4.E-05	1.E-01	1.857	1.198	1
JUN	-0.067	0.011	8.E-10	2.E-06	-0.050	0.011	5.E-06	1.E-02	1.067	0.543	1
IL13RA1	-0.066	0.011	7.E-10	2.E-06	-0.024	0.011	2.E-02	1	2.799	2.290	1

CD6	-0.066	0.011	4.E-10	1.E-06	-0.045	0.011	2.E-05	7.E-02	1.386	0.780	1
NT5E	-0.066	0.011	6.E-10	2.E-06	-0.036	0.011	8.E-04	1	1.989	1.330	1
CTSS	-0.066	0.012	5.E-08	1.E-04	-0.027	0.012	2.E-02	1	2.266	1.630	1
WARS	-0.066	0.011	7.E-10	2.E-06	-0.039	0.011	3.E-04	9.E-01	1.800	1.143	1
MTUS1	-0.066	0.011	7.E-10	2.E-06	-0.026	0.011	2.E-02	1	2.661	2.108	1
SIL1	-0.066	0.011	1.E-09	3.E-06	-0.038	0.011	5.E-04	1	1.837	1.179	1
RECK	-0.066	0.011	8.E-10	2.E-06	-0.057	0.011	2.E-07	6.E-04	0.613	0.268	1
PIGR	-0.066	0.011	8.E-10	2.E-06	-0.033	0.011	2.E-03	1	2.196	1.551	1
COL6A3	-0.066	0.010	2.E-10	5.E-07	0.008	0.010	4.E-01	1	5.224	6.757	5.E-04
ERBB2	-0.066	0.011	6.E-10	2.E-06	-0.011	0.010	3.E-01	1	3.685	3.640	7.E-01
MVK	-0.066	0.011	8.E-10	2.E-06	-0.026	0.011	1.E-02	1	2.626	2.064	1
GAS6	-0.066	0.011	8.E-10	2.E-06	-0.034	0.011	1.E-03	1	2.071	1.417	1
RET	-0.065	0.010	9.E-11	3.E-07	-0.024	0.010	2.E-02	1	2.891	2.416	1
TCL1A	-0.065	0.011	1.E-09	4.E-06	-0.042	0.011	1.E-04	3.E-01	1.550	0.917	1
CPA2	-0.065	0.011	1.E-09	4.E-06	-0.065	0.011	3.E-09	8.E-06	0.034	0.012	1
TGFA	-0.065	0.011	8.E-10	2.E-06	-0.032	0.011	3.E-03	1	2.224	1.582	1
PSIP1	-0.065	0.010	7.E-10	2.E-06	-0.039	0.011	2.E-04	6.E-01	1.716	1.065	1
MAMDC4	-0.065	0.010	3.E-10	1.E-06	-0.051	0.010	8.E-07	2.E-03	0.907	0.438	1
MICB_MICA	-0.065	0.011	2.E-09	5.E-06	-0.037	0.011	6.E-04	1	1.798	1.142	1
SIGLEC1	-0.065	0.011	9.E-10	3.E-06	-0.017	0.010	1.E-01	1	3.201	2.863	1
PAG1	-0.065	0.011	2.E-09	5.E-06	-0.038	0.011	4.E-04	1	1.751	1.097	1
SFRP4	-0.065	0.011	2.E-09	5.E-06	-0.008	0.010	5.E-01	1	3.805	3.848	4.E-01
PILRA	-0.064	0.011	1.E-09	3.E-06	-0.033	0.011	2.E-03	1	2.090	1.436	1
SIGLEC8	-0.064	0.011	3.E-09	8.E-06	-0.023	0.011	4.E-02	1	2.738	2.209	1
EFHD1	-0.064	0.010	5.E-10	1.E-06	-0.040	0.010	1.E-04	4.E-01	1.668	1.021	1
FGFR2	-0.064	0.011	2.E-09	7.E-06	-0.013	0.011	2.E-01	1	3.366	3.117	1
HSPG2	-0.064	0.011	1.E-09	3.E-06	0.002	0.010	9.E-01	1	4.504	5.175	2.E-02
TCN2	-0.064	0.011	2.E-09	6.E-06	-0.051	0.011	2.E-06	6.E-03	0.834	0.393	1
TNN	-0.064	0.011	2.E-09	6.E-06	-0.037	0.011	5.E-04	1	1.745	1.091	1
FCRL2	-0.064	0.011	2.E-09	7.E-06	-0.044	0.011	4.E-05	1.E-01	1.279	0.697	1
EPHB4	-0.064	0.011	2.E-09	5.E-06	-0.044	0.011	3.E-05	1.E-01	1.292	0.707	1
CDH6	-0.064	0.011	3.E-09	8.E-06	-0.050	0.011	4.E-06	1.E-02	0.890	0.428	1
SWAP70	-0.063	0.011	2.E-09	7.E-06	-0.036	0.011	6.E-04	1	1.787	1.131	1
BTN3A2	-0.063	0.011	3.E-09	9.E-06	-0.044	0.011	5.E-05	2.E-01	1.304	0.716	1
NDUFS6	-0.063	0.011	6.E-09	2.E-05	-0.047	0.011	2.E-05	6.E-02	1.075	0.549	1
OSCAR	-0.063	0.011	3.E-09	9.E-06	-0.044	0.011	4.E-05	1.E-01	1.241	0.669	1
GPKOW	-0.063	0.011	5.E-09	2.E-05	-0.031	0.011	5.E-03	1	2.130	1.479	1
SIGLEC10	-0.063	0.011	4.E-09	1.E-05	-0.019	0.011	7.E-02	1	2.901	2.429	1
ACAA1	-0.063	0.011	5.E-09	2.E-05	-0.030	0.011	5.E-03	1	2.143	1.493	1
PRELP	-0.063	0.010	6.E-10	2.E-06	-0.047	0.010	5.E-06	2.E-02	1.124	0.583	1
GPNMB	-0.063	0.011	7.E-09	2.E-05	-0.043	0.011	9.E-05	3.E-01	1.301	0.714	1
PRSS8	-0.063	0.010	8.E-10	2.E-06	-0.020	0.010	5.E-02	1	2.953	2.503	1
LILRB2	-0.063	0.011	4.E-09	1.E-05	-0.036	0.011	8.E-04	1	1.764	1.109	1
EGFLAM	-0.063	0.011	4.E-09	1.E-05	-0.050	0.011	3.E-06	9.E-03	0.833	0.393	1
TGFB1	-0.062	0.011	4.E-09	1.E-05	-0.028	0.011	8.E-03	1	2.303	1.673	1
IGSF8	-0.062	0.011	6.E-09	2.E-05	-0.036	0.011	9.E-04	1	1.749	1.095	1
TIMD4	-0.062	0.011	7.E-09	2.E-05	-0.033	0.011	2.E-03	1	1.928	1.269	1

CTSO	-0.062	0.011	6.E-09	2.E-05	-0.012	0.011	3.E-01	1	3.379	3.138	1
BAMBI	-0.062	0.011	1.E-08	3.E-05	-0.057	0.011	2.E-07	7.E-04	0.340	0.134	1
DDR1	-0.062	0.011	6.E-09	2.E-05	-0.046	0.011	2.E-05	5.E-02	1.029	0.518	1
FSHB	-0.062	0.011	8.E-09	2.E-05	-0.060	0.011	4.E-08	1.E-04	0.142	0.052	1
PPL	-0.062	0.011	9.E-09	3.E-05	-0.029	0.011	8.E-03	1	2.186	1.540	1
NPL	-0.062	0.010	2.E-09	5.E-06	-0.021	0.010	4.E-02	1	2.853	2.363	1
EDA2R	-0.062	0.008	5.E-14	1.E-10	-0.035	0.008	2.E-05	6.E-02	2.323	1.695	1
PTPRC	-0.062	0.011	7.E-09	2.E-05	-0.022	0.011	4.E-02	1	2.637	2.078	1
SPINK4	-0.062	0.011	6.E-09	2.E-05	-0.055	0.011	3.E-07	9.E-04	0.441	0.181	1
EGLN1	-0.062	0.010	3.E-09	8.E-06	-0.020	0.010	6.E-02	1	2.865	2.380	1
ADA2	-0.062	0.010	4.E-09	1.E-05	-0.024	0.010	2.E-02	1	2.543	1.959	1
SELPLG	-0.062	0.011	8.E-09	2.E-05	-0.030	0.011	4.E-03	1	2.057	1.402	1
LTBP3	-0.061	0.011	2.E-08	4.E-05	-0.018	0.011	9.E-02	1	2.813	2.309	1
NGRN	-0.061	0.011	8.E-09	2.E-05	-0.035	0.011	1.E-03	1	1.744	1.090	1
CCL22	-0.061	0.011	2.E-08	4.E-05	-0.028	0.011	9.E-03	1	2.142	1.492	1
OGN	-0.061	0.010	5.E-10	2.E-06	-0.018	0.010	7.E-02	1	3.118	2.739	1
CD300LF	-0.061	0.011	1.E-08	3.E-05	-0.039	0.011	3.E-04	9.E-01	1.456	0.838	1
NECTIN2	-0.061	0.011	1.E-08	4.E-05	-0.035	0.011	1.E-03	1	1.691	1.042	1
LGALS3	-0.060	0.011	1.E-08	3.E-05	-0.060	0.011	2.E-08	7.E-05	0.040	0.014	1
TNFRSF13C	-0.060	0.011	2.E-08	7.E-05	-0.038	0.011	5.E-04	1	1.451	0.833	1
AOC3	-0.060	0.011	1.E-08	4.E-05	-0.052	0.011	1.E-06	4.E-03	0.566	0.243	1
IL2RA	-0.060	0.011	2.E-08	5.E-05	-0.032	0.011	3.E-03	1	1.843	1.185	1
KLRB1	-0.060	0.011	2.E-08	5.E-05	-0.031	0.011	4.E-03	1	1.912	1.253	1
MAN1A2	-0.060	0.011	3.E-08	7.E-05	-0.061	0.011	3.E-08	8.E-05	-0.052	0.018	1
QSOX1	-0.060	0.011	2.E-08	7.E-05	-0.021	0.011	5.E-02	1	2.556	1.975	1
LRIG3	-0.060	0.011	3.E-08	8.E-05	-0.046	0.011	3.E-05	7.E-02	0.921	0.447	1
POLR2F	-0.060	0.011	1.E-08	4.E-05	-0.014	0.010	2.E-01	1	3.090	2.699	1
SAA4	-0.060	0.011	1.E-08	4.E-05	-0.041	0.011	1.E-04	4.E-01	1.265	0.686	1
MARCO	-0.060	0.011	3.E-08	8.E-05	-0.023	0.011	3.E-02	1	2.425	1.816	1
SERPINI1	-0.060	0.011	3.E-08	1.E-04	-0.053	0.011	1.E-06	3.E-03	0.408	0.166	1
KYNU	-0.060	0.011	2.E-08	5.E-05	-0.013	0.010	2.E-01	1	3.112	2.730	1
LPCAT2	-0.060	0.011	2.E-08	6.E-05	-0.043	0.011	6.E-05	2.E-01	1.082	0.554	1
ECHDC3	-0.060	0.011	2.E-08	5.E-05	-0.027	0.011	1.E-02	1	2.163	1.516	1
C7	-0.059	0.010	1.E-08	4.E-05	-0.042	0.011	8.E-05	2.E-01	1.191	0.631	1
MDGA1	-0.059	0.011	4.E-08	1.E-04	-0.060	0.011	4.E-08	1.E-04	-0.044	0.016	1
TNC	-0.059	0.011	4.E-08	1.E-04	-0.070	0.011	1.E-10	4.E-07	-0.722	0.328	1
CREG1	-0.059	0.011	6.E-08	2.E-04	-0.015	0.011	2.E-01	1	2.881	2.402	1
ORM1	-0.059	0.010	1.E-08	4.E-05	-0.046	0.010	1.E-05	4.E-02	0.891	0.429	1
TNFRSF21	-0.059	0.011	3.E-08	1.E-04	-0.036	0.011	8.E-04	1	1.509	0.882	1
MFAP4	-0.059	0.010	2.E-08	6.E-05	-0.028	0.010	7.E-03	1	2.038	1.382	1
ZHX2	-0.059	0.010	2.E-08	6.E-05	-0.024	0.010	2.E-02	1	2.341	1.716	1
ANXA10	-0.058	0.011	4.E-08	1.E-04	-0.050	0.011	4.E-06	1.E-02	0.563	0.241	1
PILRB	-0.058	0.011	5.E-08	1.E-04	-0.032	0.011	3.E-03	1	1.740	1.087	1
KLF4	-0.058	0.011	6.E-08	2.E-04	-0.029	0.011	7.E-03	1	1.923	1.264	1
A1BG	-0.058	0.011	4.E-08	1.E-04	-0.046	0.011	1.E-05	4.E-02	0.776	0.359	1
PDGFRA	-0.058	0.011	5.E-08	1.E-04	-0.040	0.011	2.E-04	5.E-01	1.182	0.625	1
SULT2A1	-0.058	0.011	6.E-08	2.E-04	-0.004	0.010	7.E-01	1	3.586	3.474	1.E+00

APBB1IP	-0.058	0.011	4.E-08	1.E-04	-0.018	0.011	9.E-02	1	2.674	2.126	1
TREM2	-0.058	0.010	8.E-09	2.E-05	-0.028	0.010	5.E-03	1	2.083	1.429	1
DTX3	-0.058	0.010	2.E-08	7.E-05	-0.042	0.010	7.E-05	2.E-01	1.094	0.563	1
BTN2A1	-0.058	0.011	6.E-08	2.E-04	-0.030	0.011	5.E-03	1	1.857	1.199	1
GALNT7	-0.057	0.011	9.E-08	3.E-04	-0.053	0.011	1.E-06	4.E-03	0.302	0.118	1
TIMP2	-0.057	0.011	5.E-08	1.E-04	-0.028	0.011	8.E-03	1	1.983	1.325	1
SELE	-0.057	0.010	4.E-08	1.E-04	-0.004	0.010	7.E-01	1	3.670	3.615	7.E-01
CD8A	-0.057	0.011	7.E-08	2.E-04	-0.031	0.011	3.E-03	1	1.719	1.068	1
DNPEP	-0.057	0.011	5.E-08	2.E-04	-0.039	0.011	3.E-04	8.E-01	1.247	0.673	1
DKK4	-0.057	0.011	1.E-07	3.E-04	-0.043	0.011	8.E-05	2.E-01	0.938	0.458	1
CD83	-0.057	0.011	9.E-08	3.E-04	-0.024	0.011	3.E-02	1	2.210	1.566	1
FLT3LG	-0.057	0.010	3.E-08	8.E-05	-0.034	0.010	1.E-03	1	1.606	0.966	1
HJV	-0.057	0.010	4.E-08	1.E-04	-0.015	0.010	2.E-01	1	2.875	2.394	1
TYRO3	-0.057	0.011	1.E-07	4.E-04	-0.034	0.011	1.E-03	1	1.471	0.850	1
LRG1	-0.057	0.010	6.E-08	2.E-04	-0.058	0.011	5.E-08	1.E-04	-0.084	0.030	1
IFNG	-0.057	0.011	1.E-07	4.E-04	-0.048	0.011	9.E-06	3.E-02	0.541	0.230	1
IL18BP	-0.057	0.011	7.E-08	2.E-04	-0.033	0.011	2.E-03	1	1.573	0.936	1
HLA_E	-0.057	0.011	9.E-08	3.E-04	-0.035	0.011	9.E-04	1	1.409	0.799	1
CD27	-0.057	0.010	7.E-08	2.E-04	-0.032	0.011	2.E-03	1	1.629	0.985	1
XCL1	-0.056	0.011	1.E-07	4.E-04	-0.024	0.011	2.E-02	1	2.127	1.476	1
SIGLEC5	-0.056	0.011	2.E-07	5.E-04	-0.036	0.011	8.E-04	1	1.309	0.720	1
MPO	-0.056	0.011	1.E-07	3.E-04	-0.023	0.011	3.E-02	1	2.207	1.563	1
SNRPB2	-0.056	0.010	7.E-08	2.E-04	-0.023	0.010	3.E-02	1	2.248	1.609	1
SERPINA9	-0.056	0.011	2.E-07	6.E-04	-0.022	0.011	4.E-02	1	2.243	1.604	1
CILP	-0.056	0.010	9.E-08	3.E-04	-0.009	0.010	4.E-01	1	3.164	2.808	1
GAS2	-0.056	0.011	2.E-07	5.E-04	-0.021	0.011	6.E-02	1	2.338	1.713	1
TNFSF13	-0.056	0.010	4.E-08	1.E-04	-0.046	0.010	7.E-06	2.E-02	0.658	0.292	1
CLGN	-0.056	0.011	2.E-07	7.E-04	-0.039	0.011	4.E-04	1	1.114	0.576	1
SRPX	-0.056	0.010	1.E-08	4.E-05	-0.046	0.010	4.E-06	1.E-02	0.718	0.326	1
ZBP1	-0.056	0.011	2.E-07	6.E-04	-0.043	0.011	6.E-05	2.E-01	0.811	0.379	1
KRT19	-0.056	0.011	1.E-07	4.E-04	-0.050	0.011	3.E-06	9.E-03	0.385	0.155	1
CCL18	-0.056	0.011	2.E-07	5.E-04	-0.025	0.011	2.E-02	1	2.068	1.413	1
TREH	-0.055	0.011	1.E-07	4.E-04	-0.014	0.010	2.E-01	1	2.827	2.328	1
INHBC	-0.055	0.010	1.E-07	3.E-04	0.006	0.010	5.E-01	1	4.247	4.664	6.E-02
LGALS1	-0.055	0.011	2.E-07	5.E-04	0.002	0.010	8.E-01	1	3.912	4.038	3.E-01
GM2A	-0.055	0.010	1.E-07	3.E-04	-0.015	0.010	2.E-01	1	2.748	2.223	1
SART1	-0.055	0.011	2.E-07	7.E-04	-0.025	0.011	2.E-02	1	2.006	1.348	1
PREB	-0.055	0.011	4.E-07	1.E-03	-0.041	0.011	2.E-04	6.E-01	0.927	0.451	1
SERPINB8	-0.055	0.011	2.E-07	6.E-04	-0.022	0.011	4.E-02	1	2.225	1.584	1
CCDC80	-0.055	0.010	6.E-08	2.E-04	-0.008	0.010	4.E-01	1	3.260	2.953	1
GALNT3	-0.055	0.011	4.E-07	1.E-03	-0.042	0.011	1.E-04	3.E-01	0.806	0.377	1
CCL16	-0.055	0.011	2.E-07	6.E-04	-0.007	0.010	5.E-01	1	3.246	2.931	1
F7	-0.054	0.010	2.E-07	6.E-04	-0.026	0.011	1.E-02	1	1.927	1.268	1
PADI2	-0.054	0.011	6.E-07	2.E-03	-0.042	0.011	1.E-04	4.E-01	0.808	0.378	1
BCAT1	-0.054	0.010	2.E-07	7.E-04	-0.035	0.011	9.E-04	1	1.264	0.686	1
NPPB	-0.054	0.010	2.E-07	6.E-04	-0.047	0.011	9.E-06	3.E-02	0.484	0.202	1
ERN1	-0.054	0.011	3.E-07	9.E-04	-0.033	0.011	2.E-03	1	1.391	0.785	1

PDCD1	-0.054	0.011	5.E-07	2.E-03	-0.035	0.011	1.E-03	1	1.226	0.657	1
CLEC6A	-0.054	0.011	6.E-07	2.E-03	-0.033	0.011	2.E-03	1	1.366	0.765	1
C8B	-0.054	0.011	4.E-07	1.E-03	-0.045	0.011	3.E-05	9.E-02	0.575	0.248	1
ATRN	-0.054	0.011	4.E-07	1.E-03	-0.015	0.011	2.E-01	1	2.606	2.039	1
IFNGR2	-0.054	0.011	6.E-07	2.E-03	-0.021	0.011	5.E-02	1	2.119	1.468	1
TNFRSF13B	-0.054	0.011	4.E-07	1.E-03	-0.019	0.011	8.E-02	1	2.346	1.722	1
OXT	-0.054	0.010	3.E-07	8.E-04	0.011	0.010	3.E-01	1	4.453	5.073	2.E-02
SOX9	-0.053	0.011	7.E-07	2.E-03	-0.045	0.011	4.E-05	1.E-01	0.561	0.241	1
FCN1	-0.053	0.011	4.E-07	1.E-03	-0.025	0.011	2.E-02	1	1.935	1.276	1
FAP	-0.053	0.011	6.E-07	2.E-03	-0.025	0.011	2.E-02	1	1.899	1.240	1
PDIA3	-0.053	0.011	8.E-07	2.E-03	-0.038	0.011	5.E-04	1	1.017	0.510	1
CCL19	-0.053	0.011	6.E-07	2.E-03	-0.017	0.011	1.E-01	1	2.376	1.756	1
SERPINA11	-0.053	0.010	2.E-07	7.E-04	-0.055	0.010	1.E-07	3.E-04	-0.152	0.056	1
CD46	-0.053	0.011	9.E-07	3.E-03	-0.032	0.011	3.E-03	1	1.378	0.774	1
ITGAL	-0.053	0.011	9.E-07	3.E-03	-0.024	0.011	3.E-02	1	1.893	1.233	1
NMNAT1	-0.053	0.010	4.E-07	1.E-03	-0.026	0.010	1.E-02	1	1.825	1.167	1
AKR1B10	-0.053	0.011	1.E-06	3.E-03	-0.026	0.011	1.E-02	1	1.706	1.055	1
AKR7L	-0.053	0.011	1.E-06	3.E-03	-0.015	0.011	2.E-01	1	2.456	1.852	1
CTSH	-0.053	0.011	1.E-06	3.E-03	-0.036	0.011	1.E-03	1	1.090	0.559	1
TINAGL1	-0.052	0.011	7.E-07	2.E-03	-0.043	0.011	6.E-05	2.E-01	0.635	0.280	1
HHEX	-0.052	0.010	5.E-07	1.E-03	-0.027	0.011	1.E-02	1	1.731	1.078	1
CDH17	-0.052	0.011	1.E-06	3.E-03	-0.051	0.011	3.E-06	9.E-03	0.098	0.035	1
MASP1	-0.052	0.011	1.E-06	4.E-03	-0.014	0.011	2.E-01	1	2.493	1.898	1
CNTN1	-0.052	0.011	1.E-06	3.E-03	-0.056	0.011	2.E-07	5.E-04	-0.286	0.111	1
TNFRSF14	-0.052	0.011	9.E-07	3.E-03	-0.020	0.011	6.E-02	1	2.153	1.505	1
TGFBI	-0.052	0.011	1.E-06	4.E-03	-0.032	0.011	3.E-03	1	1.290	0.706	1
TK1	-0.052	0.011	1.E-06	4.E-03	-0.037	0.011	7.E-04	1	0.984	0.488	1
PTN	-0.052	0.010	7.E-07	2.E-03	-0.022	0.010	3.E-02	1	1.986	1.327	1
ACVRL1	-0.052	0.010	8.E-07	2.E-03	-0.018	0.010	8.E-02	1	2.246	1.607	1
WDR46	-0.051	0.011	1.E-06	3.E-03	-0.027	0.011	1.E-02	1	1.620	0.978	1
LETM1	-0.051	0.011	1.E-06	4.E-03	-0.034	0.011	1.E-03	1	1.131	0.589	1
SEMA4C	-0.051	0.011	2.E-06	5.E-03	-0.044	0.011	5.E-05	1.E-01	0.455	0.188	1
TNFRSF9	-0.051	0.011	1.E-06	4.E-03	-0.017	0.011	1.E-01	1	2.302	1.671	1
CLEC7A	-0.051	0.011	1.E-06	4.E-03	-0.030	0.011	5.E-03	1	1.411	0.801	1
EDIL3	-0.051	0.011	2.E-06	5.E-03	-0.068	0.011	2.E-10	5.E-07	-1.166	0.614	1
CLEC3B	-0.051	0.010	1.E-06	4.E-03	-0.037	0.011	6.E-04	1	0.948	0.465	1
NTPROBNP	-0.051	0.010	2.E-07	6.E-04	-0.047	0.010	2.E-06	6.E-03	0.251	0.096	1
ADAMTS1	-0.050	0.011	3.E-06	8.E-03	-0.035	0.011	1.E-03	1	1.037	0.523	1
SSC5D	-0.050	0.010	2.E-06	4.E-03	-0.003	0.010	8.E-01	1	3.253	2.942	1
AKR1C4	-0.050	0.011	4.E-06	1.E-02	-0.027	0.011	1.E-02	1	1.504	0.877	1
NOTCH3	-0.050	0.010	5.E-07	1.E-03	-0.044	0.010	1.E-05	4.E-02	0.414	0.168	1
ANGPTL2	-0.050	0.011	2.E-06	7.E-03	0.007	0.010	5.E-01	1	3.877	3.977	3.E-01
DMD	-0.050	0.011	2.E-06	7.E-03	-0.029	0.011	6.E-03	1	1.392	0.786	1
ASAH1	-0.050	0.011	3.E-06	8.E-03	-0.023	0.011	3.E-02	1	1.791	1.135	1
LMOD1	-0.050	0.010	3.E-07	7.E-04	-0.051	0.010	2.E-07	5.E-04	-0.097	0.035	1
CSTB	-0.050	0.011	2.E-06	7.E-03	-0.012	0.011	3.E-01	1	2.535	1.949	1
CCL14	-0.050	0.011	3.E-06	8.E-03	-0.032	0.011	3.E-03	1	1.193	0.633	1

SPINK1	-0.050	0.011	2.E-06	7.E-03	-0.051	0.011	2.E-06	6.E-03	-0.071	0.025	1
LDLR	-0.050	0.010	1.E-06	3.E-03	-0.004	0.010	7.E-01	1	3.171	2.818	1
CSF2	-0.050	0.011	4.E-06	1.E-02	-0.028	0.011	1.E-02	1	1.417	0.805	1
CDH1	-0.050	0.011	5.E-06	1.E-02	-0.022	0.011	5.E-02	1	1.828	1.170	1
RPL14	-0.050	0.011	4.E-06	1.E-02	-0.015	0.011	2.E-01	1	2.313	1.683	1
NEB	-0.050	0.011	4.E-06	1.E-02	-0.029	0.011	7.E-03	1	1.330	0.736	1
CCN4	-0.050	0.011	3.E-06	8.E-03	-0.011	0.011	3.E-01	1	2.585	2.012	1
TEK	-0.049	0.011	4.E-06	1.E-02	-0.029	0.011	7.E-03	1	1.323	0.731	1
IGF2R	-0.049	0.011	4.E-06	1.E-02	-0.016	0.011	1.E-01	1	2.194	1.549	1
LAMP1	-0.049	0.011	4.E-06	1.E-02	-0.020	0.011	7.E-02	1	1.940	1.281	1
IFNGR1	-0.049	0.011	4.E-06	1.E-02	-0.039	0.011	4.E-04	1	0.694	0.312	1
SNED1	-0.049	0.011	5.E-06	1.E-02	-0.040	0.011	3.E-04	8.E-01	0.624	0.274	1
NCR1	-0.049	0.011	5.E-06	2.E-02	-0.027	0.011	1.E-02	1	1.454	0.836	1
EFCAB14	-0.049	0.010	3.E-06	1.E-02	-0.022	0.011	4.E-02	1	1.786	1.130	1
RTN4R	-0.049	0.010	2.E-06	7.E-03	0.023	0.010	2.E-02	1	5.050	6.354	1.E-03
NFATC3	-0.049	0.011	7.E-06	2.E-02	-0.021	0.011	5.E-02	1	1.799	1.143	1
ESM1	-0.049	0.010	3.E-06	9.E-03	-0.064	0.011	2.E-09	5.E-06	-1.012	0.507	1
LIF	-0.049	0.011	9.E-06	2.E-02	-0.030	0.011	6.E-03	1	1.178	0.622	1
REN	-0.049	0.011	5.E-06	2.E-02	-0.020	0.011	6.E-02	1	1.898	1.239	1
DDAH1	-0.049	0.011	6.E-06	2.E-02	-0.017	0.011	1.E-01	1	2.055	1.399	1
HRG	-0.048	0.011	6.E-06	2.E-02	-0.021	0.011	5.E-02	1	1.784	1.129	1
MMP7	-0.048	0.010	3.E-06	9.E-03	-0.023	0.010	3.E-02	1	1.750	1.097	1
DUSP29	-0.048	0.011	7.E-06	2.E-02	-0.029	0.011	8.E-03	1	1.272	0.692	1
FGR	-0.048	0.010	4.E-06	1.E-02	-0.029	0.011	7.E-03	1	1.322	0.730	1
COL18A1	-0.048	0.010	3.E-06	9.E-03	-0.019	0.010	6.E-02	1	1.984	1.325	1
VWF	-0.048	0.011	5.E-06	1.E-02	-0.025	0.011	2.E-02	1	1.538	0.906	1
CXADR	-0.048	0.011	7.E-06	2.E-02	-0.031	0.011	4.E-03	1	1.110	0.574	1
BGN	-0.048	0.011	9.E-06	3.E-02	-0.050	0.011	5.E-06	1.E-02	-0.137	0.050	1
CWC15	-0.048	0.011	5.E-06	1.E-02	-0.022	0.011	4.E-02	1	1.779	1.124	1
LRIG1	-0.048	0.011	7.E-06	2.E-02	-0.036	0.011	9.E-04	1	0.781	0.361	1
NAGPA	-0.048	0.010	5.E-06	2.E-02	-0.024	0.011	2.E-02	1	1.560	0.925	1
TGOLN2	-0.048	0.010	6.E-06	2.E-02	-0.029	0.011	7.E-03	1	1.282	0.699	1
PRUNE2	-0.047	0.011	1.E-05	3.E-02	-0.025	0.011	2.E-02	1	1.470	0.849	1
PRCP	-0.047	0.011	8.E-06	2.E-02	0.014	0.010	2.E-01	1	4.114	4.410	1.E-01
VAMP5	-0.047	0.011	1.E-05	4.E-02	-0.032	0.011	4.E-03	1	1.017	0.510	1
LRCH4	-0.047	0.010	5.E-06	2.E-02	-0.024	0.010	2.E-02	1	1.600	0.960	1
ZBTB17	-0.047	0.011	1.E-05	3.E-02	-0.022	0.011	4.E-02	1	1.686	1.037	1
CD164	-0.047	0.011	1.E-05	3.E-02	-0.027	0.011	1.E-02	1	1.348	0.750	1
CEBPA	-0.047	0.011	1.E-05	4.E-02	-0.030	0.011	5.E-03	1	1.075	0.549	1
PCDH9	-0.047	0.011	1.E-05	3.E-02	-0.021	0.011	5.E-02	1	1.736	1.083	1
LTBR	-0.047	0.011	1.E-05	3.E-02	-0.015	0.011	1.E-01	1	2.101	1.448	1
SEPTIN8	-0.047	0.010	6.E-06	2.E-02	-0.017	0.010	1.E-01	1	2.031	1.374	1
RBP7	-0.047	0.010	6.E-06	2.E-02	0.000	0.010	1.E+00	1	3.215	2.884	1
NBL1	-0.047	0.010	7.E-06	2.E-02	-0.018	0.010	9.E-02	1	1.952	1.293	1
ERBB3	-0.047	0.010	5.E-06	1.E-02	-0.056	0.010	5.E-08	1.E-04	-0.661	0.293	1
CRHBP	-0.046	0.011	1.E-05	4.E-02	0.000	0.011	1.E+00	1	3.108	2.725	1
CD109	-0.046	0.011	2.E-05	5.E-02	-0.024	0.011	3.E-02	1	1.470	0.849	1

THOP1	-0.046	0.011	1.E-05	3.E-02	-0.011	0.011	3.E-01	1	2.352	1.729	1
TCTN3	-0.046	0.011	1.E-05	3.E-02	-0.008	0.011	5.E-01	1	2.594	2.023	1
SCLY	-0.046	0.011	1.E-05	3.E-02	0.005	0.010	7.E-01	1	3.439	3.234	1
FLT4	-0.046	0.011	1.E-05	4.E-02	-0.017	0.011	1.E-01	1	1.928	1.268	1
STAB2	-0.046	0.010	1.E-05	3.E-02	-0.004	0.010	7.E-01	1	2.841	2.347	1
CA12	-0.046	0.011	1.E-05	4.E-02	-0.025	0.011	2.E-02	1	1.422	0.809	1
KIF1C	-0.046	0.011	2.E-05	5.E-02	-0.023	0.011	4.E-02	1	1.544	0.912	1
OSM	-0.046	0.011	2.E-05	5.E-02	-0.013	0.011	2.E-01	1	2.213	1.570	1
MNDA	-0.046	0.010	7.E-06	2.E-02	-0.032	0.010	2.E-03	1	0.975	0.482	1
COL1A1	-0.046	0.011	2.E-05	5.E-02	-0.044	0.011	5.E-05	1.E-01	0.118	0.043	1
TFF3	-0.044	0.010	2.E-05	4.E-02	-0.036	0.010	5.E-04	1	0.574	0.247	1
IGFBP4	-0.044	0.010	1.E-05	3.E-02	-0.004	0.010	7.E-01	1	2.811	2.307	1

Linear regression analyses to estimate associations of SD change in inverse rank normalized circulating protein levels per 1 SD change in CAG and GGC trinucleotide repeat lengths or total testosterone which are independent with each other, adjusted for age, 10 ancestry PCs, assessment center, SHBG, fasting time, and batch, with or without BMI, were performed in European-ancestry male participants in the UK Biobank (n = 14,353). CAG and GGC repeat lengths were quantified from WES data. For each protein, Z score was calculated from the difference between betas (beta with adjustment for BMI - beta without adjustment for BMI) and standard errors and converted into P value. P values were corrected for the 2,921 protein levels with the Bonferroni method. Only those showing an association with a repeat length or total testosterone without adjustment for BMI with statistical significance (corrected P value <0.05) are listed.
